# Oleanolic acid improved intestinal immune function by activating and potentiating bile acids receptor signaling in *E. coli*-challenged piglets

**DOI:** 10.1186/s40104-024-01037-0

**Published:** 2024-05-18

**Authors:** Chenyu Xue, Hongpeng Jia, Rujing Cao, Wenjie Cai, Weichen Hong, Jianing Tu, Songtao Wang, Qianzhi Jiang, Chongpeng Bi, Anshan Shan, Na Dong

**Affiliations:** The Laboratory of Molecular Nutrition and Immunity, College of Animal Science and Technology, Northeast Agricultural University, Harbin, P. R. China

**Keywords:** Bile acid receptors, Enterotoxigenic *Escherichia coli*, Intestinal innate immunity, Oleanolic acid

## Abstract

**Background:**

Infection with pathogenic bacteria during nonantibiotic breeding is one of the main causes of animal intestinal diseases. Oleanolic acid (OA) is a pentacyclic triterpene that is ubiquitous in plants. Our previous work demonstrated the protective effect of OA on intestinal health, but the underlying molecular mechanisms remain unclear. This study investigated whether dietary supplementation with OA can prevent diarrhea and intestinal immune dysregulation caused by enterotoxigenic *Escherichia coli* (ETEC) in piglets. The key molecular role of bile acid receptor signaling in this process has also been explored.

**Results:**

Our results demonstrated that OA supplementation alleviated the disturbance of bile acid metabolism in ETEC-infected piglets (*P* < 0.05). OA supplementation stabilized the composition of the bile acid pool in piglets by regulating the enterohepatic circulation of bile acids and significantly increased the contents of UDCA and CDCA in the ileum and cecum (*P* < 0.05). This may also explain why OA can maintain the stability of the intestinal microbiota structure in ETEC-challenged piglets. In addition, as a natural ligand of bile acid receptors, OA can reduce the severity of intestinal inflammation and enhance the strength of intestinal epithelial cell antimicrobial programs through the bile acid receptors TGR5 and FXR (*P* < 0.05). Specifically, OA inhibited NF-κB-mediated intestinal inflammation by directly activating TGR5 and its downstream cAMP-PKA-CREB signaling pathway (*P* < 0.05). Furthermore, OA enhanced CDCA-mediated MEK-ERK signaling in intestinal epithelial cells by upregulating the expression of FXR (*P* < 0.05), thereby upregulating the expression of endogenous defense molecules in intestinal epithelial cells.

**Conclusions:**

In conclusion, our findings suggest that OA-mediated regulation of bile acid metabolism plays an important role in the innate immune response, which provides a new diet-based intervention for intestinal diseases caused by pathogenic bacterial infections in piglets.

**Graphical Abstract:**

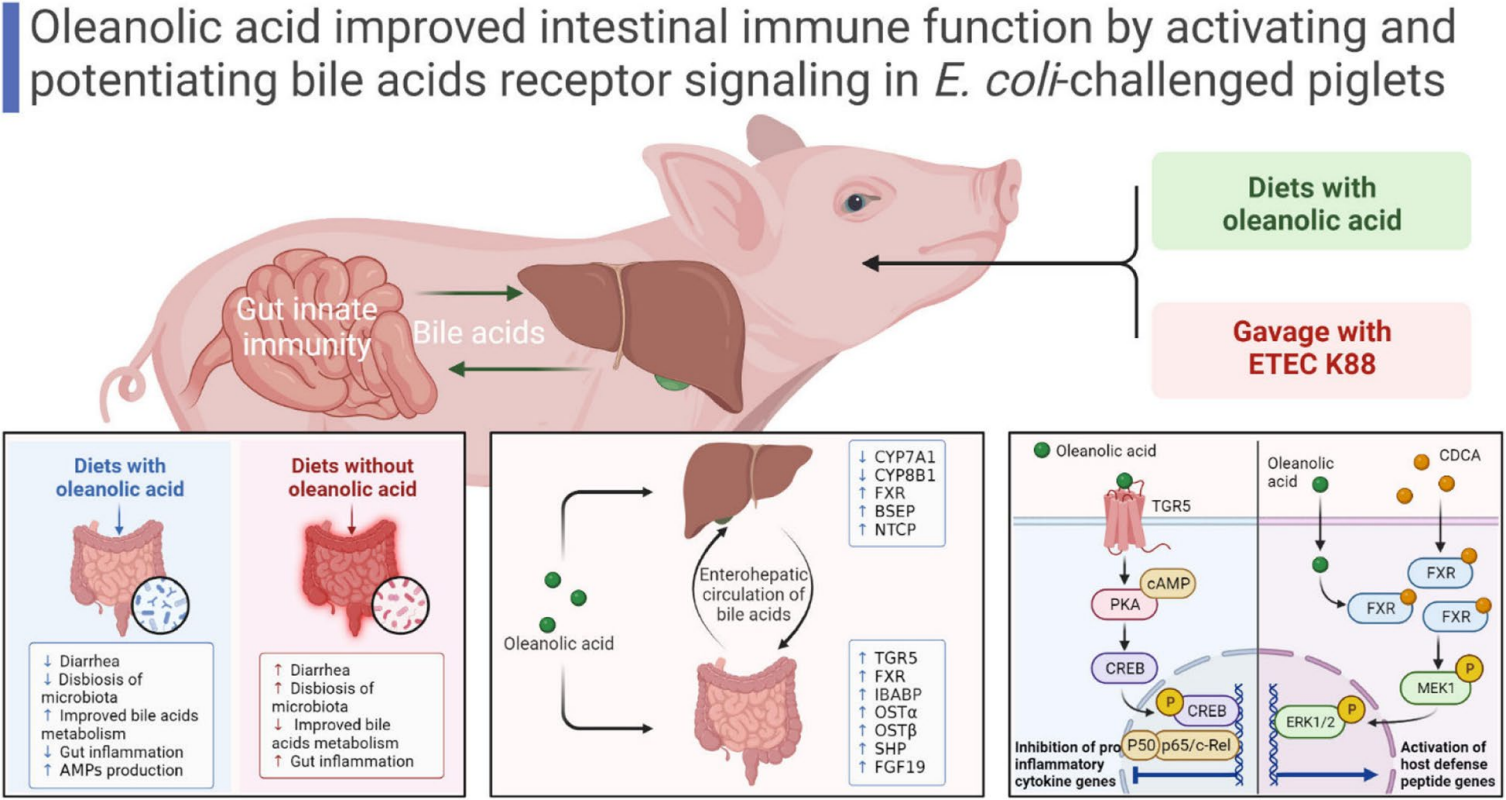

**Supplementary Information:**

The online version contains supplementary material available at 10.1186/s40104-024-01037-0.

## Background

The intestinal mucosal immune system is the largest mucosal immune organ in the body and plays a crucial role in the recognition and clearance of pathogens and harmful substances; this process is intimately linked to the overall health of animals [1]. Therefore, it is important to maintain the proper function of the intestinal immune system in animal production. For instance, in swine production, the digestive and immune functions of weaned piglet intestines are not fully developed, resulting in limited adaptability to complex environments and increased susceptibility to various pathogens [2]. Extensive research has shown that during this stage, piglets have a higher incidence of diarrhea and intestinal inflammation, leading to piglet mortality and resulting in significant economic losses [3–5]. Traditionally, feed antibiotics, including those responsible for piglet diarrhea, have been employed as preventive measures to control and manage bacterial infections [6]. However, mounting evidence highlighting the potential for antibiotic resistance development and transmission has prompted many countries, including China, to gradually begin banning the use of in-feed antibiotics in livestock production [7]. It is becoming increasingly important to find effective and safe antibiotic substitutes to reduce the risk of intestinal disease in piglets at the farm level.

Alternatives to antibiotics include natural small molecules, probiotics, prebiotics, and synbiotics, to name a few, of which the use of natural small molecules to prevent diarrhea is a good option. Oleanolic acid (OA) is a pentacyclic triterpenoid compound found in many plants, including fruits, vegetables, and medicinal herbs [8]. Recent studies on OA have revealed its wide range of pharmacological effects, including antioxidant [9], anti-inflammatory [10], hepatoprotective [11], antimicrobial [12], and anticancer properties [13]. These discoveries have sparked considerable interest in exploring the potential applications of OA in multiple fields. Multiple reports demonstrate the potential of OA as a therapeutic agent for a variety of diseases, including liver diseases [14], cardiovascular conditions [15], cancer [16], and skin disorders [17]. Our recent study demonstrated the protective effect of OA on intestinal immune homeostasis in a rodent model, suggesting the potential of OA as an alternative to antibiotics [18, 19]. The mechanism underlying this effect involves the regulatory function of OA on the gut-liver axis.

Bile acids, as important components of the gut-liver axis cycle, are involved in the regulation of several key cellular processes through the activation of multiple receptors. Bile acids, as signaling molecules, can participate in regulating a range of crucial cellular processes by activating multiple receptors. These processes include bile acid metabolism, glucose homeostasis, lipid metabolism, energy expenditure, intestinal motility, and immune cell function [20]. Known bile acid receptors can be classified into two major categories: nuclear receptors and membrane receptors. Nuclear receptors include farnesoid X receptor (FXR), vitamin D receptor (VDR), pregnane X receptor (PXR), and constitutive androstane receptor (CAR). Membrane receptors primarily consist of G protein-coupled receptor 5 (TGR5) and sphingosine-1-phosphate receptor 2 (S1PR2) [21]. These receptors are widely expressed in various tissues throughout the body. Extensive research has been conducted on the FXR and TGR5 receptors, which play a role in regulating bile acid metabolism, glucose and lipid metabolism, and even innate immune responses [22]. The pharmacological activity of OA may be mediated by bile acid receptors. For example, OA has been demonstrated to be a potent agonist of TGR5 [23]. Activation of TGR5 by OA can improve glucose levels in diabetic rats and alleviate hepatic steatosis and lipid peroxidation, thereby inhibiting the development of liver fibrosis [24]. OA also ameliorated irinotecan-induced colitis in rats by activating TGR5 and inhibiting the NOD-like receptor thermal protein domain associated protein 3 (NLRP3) inflammasome [25]. However, the specific regulatory effects of OA on the bile acid receptor signaling cascade in the intestine of weaned piglets have not been determined.

In the present study, we hypothesized that OA could modulate the innate immune response of intestinal epithelial cells via bile acid receptors and thus protect against ETEC-induced intestinal injury in piglets. Therefore, our study aimed to investigate the regulatory role of OA in ameliorating the inflammatory and antimicrobial responses of intestinal epithelial cells upon invasion by pathogens. This study provides an important theoretical basis for the use of OA as a new nutritional intervention to improve intestinal health in young animals.

## Materials and methods

### Ethics statement

A total of 32 male weaned piglets (Duroc-Landrace-Yorkshire; initial weight: 8.81 ± 0.42 kg; 28 days old) were used in this study. The basal diet (Additional file 1: Table S1) was formulated following the recommendations of the Nutrient Requirements of Swine (China, GB/T 39235–2020) [26] for piglets weighing 8 to 25 kg. Piglets were individually housed in metabolic cages (cage size: 1.4 m × 0.7 m × 0.8 m), allowing them to freely access food and water. All metabolic cages were evenly distributed among four isolated rooms to prevent cross-contamination of ETEC. The temperature of the barn was controlled between 23 and 25 °C, and the relative humidity was 50%–70%. The procedures of this research were approved by the Northeast Agricultural University (NEAU) Institutional Animal Care and Use Committee, and the ethical treatment of animals was approved by the NEAU Animal Welfare Committee Protocol (NEAU-[2013]-9).

### Animals and treatment

The 32 piglets were randomly divided into 4 treatment groups with 8 replicates of 1 pig each. The 4 treatment groups included: 1) control group (CON), piglets were fed with a basal diet; 2) ETEC challenge group (ETEC), piglets were fed with a basal diet and challenged by ETEC; 3) OA prevention group (OA + ETEC), piglets were fed with a basal diet containing 0.01% OA (purity ≥ 98%, Beijing Solarbio Science & Technology Co., Ltd., Beijing, China) and challenged by ETEC; and 4) OA control group (OA), piglets were fed with a basal diet containing 0.01% OA. The trial lasted for 14 d. On d 10, piglets in ETEC group and OA + ETEC group were orally administered 20 mL of LB culture medium containing 5 × 10^9^ CFU/mL ETEC at logarithmic growth stage for 4 consecutive days. CON group and OA group received an equal amount of culture medium orally. *Escherichia coli* strain O149:K91:K88ac was purchased from China Veterinary Culture Collection Center (CVCC, Beijing, China).

The procedures of this study were adapted from the research methodology summarized by Luise et al. [27]. The severity of diarrhea in piglets was assessed by the diarrhea index. The diarrhea index for each piglet was visually evaluated by 2 independent evaluators each day after the ETEC challenge, with the score ranging from 1 to 5 (1 = hard and dry feces, 2 = well-formed firm feces, 3 = formed feces, 4 = pasty feces, and 5 = liquid diarrhea). A diarrhea index > 3 was defined as a clinical sign of diarrhea.

### Sample collection

At the end of the experiment (d 15), all piglets were stunned by electric shock and killed by jugular bloodletting. Blood samples were obtained through jugular vein puncture, followed by centrifugation at 3000 × *g* for 10 min at 4 °C to obtain serum. The serum was stored at −20 °C for further analysis. Once the piglets showed no signs of life, the abdominal cavity was opened with a scalpel to fully expose the internal organs. Subsequently, portal vein blood, liver, intestinal tissue, and chyme were collected, then frozen by immersion in liquid nitrogen, and stored at −80 °C until analysis. Fresh tissue intended for hematoxylin and eosin (HE) staining was preserved in 4% paraformaldehyde.

### Measurement by ELISA

Concentrations of tumor necrosis factor (TNF-α), interleukin-6 (IL-6), interleukin-1β (IL-1β), lipopolysaccharides (LPS), D-lactate and intestinal-type fatty acid binding protein (iFABP) in serum and cAMP in cell were determined using the instructions in the commercially available ELISA kits (Abcam Limited, MA, USA).

### Histological staining

After fixation in 4% paraformaldehyde, liver, and small intestine samples were embedded in paraffin and cut into 4 μm sections. The tissue sections were subjected to deparaffinization and hydration using a series of graded alcohols. Subsequently, the sections were stained. Following a rinse with distilled water, the slides were dehydrated in alcohol and then placed in a clearing solution of xylene. Tissue morphology was observed using a microscope (B80i, Nikon Corporation, Tokyo, Japan) with image acquisition software. The histological scoring of the small intestine was determined based on epithelial lesions and inflammation infiltration, following the same criteria as previously reported [28].

### Immunofluorescence

TGR5 detection was performed on paraffin-embedded sections of ileum tissue following the standard protocol. The tissue sections were deparaffinized in xylene, followed by rehydration in a series of ethanol and distilled water. Subsequently, they were immersed in ethylenediaminetetraacetic acid (EDTA) buffer (pH 8.0, Beyotime Biotechnology Co., Ltd., Shanghai, China) for antigen retrieval. Afterward, the slides were washed 3 times with phosphate-buffered saline (PBS, pH 7.4) to remove any residual buffer. After the sections were slightly dry, circles were drawn on the tissue using Liquid Blocker Super PAP Pen (Beyotime) to prevent antibody flow away, and then blocked with 3% BSA for 30 min. After gently removing the blocking solution, rabbit anti-TGR5 antibody (PA5–34261, 1:200 dilution, Thermo Fisher Scientific, MA, USA) was added to the sections and incubated overnight at 4 °C. Subsequently, the phosphate buffered saline (PBS)-washed samples were incubated with secondary antibodies conjugated to Alexa Fluor 594 (1:1000 dilution, Thermo Fisher Scientific) for 1 h at 37 °C. Within the circle, 4′,6-diamidino-2-phenylindole (DAPI) (MilliporeSigma, Burlington, MA, USA) nuclear stain was added for nuclear staining and incubated in the dark at room temperature for 10 min. Finally, slides were washed with PBS and sealed with anti-fluorescence quenching sealer (Servicebio Technology Co., Ltd., Wuhan, China). All slides were observed, and images were collected by fluorescence microscopy (Eclipse C1, Nikon, Tokyo, Japan).

### RNA isolation and RT-PCR

Total RNA was extracted from the liver, jejunum, and ileum using TRIzol reagent (Invitrogen, Carlsbad, CA, USA). cDNA was synthesized from 1 μg of RNA using the PrimeScript RT kit (Takara Biotechnology Co., Ltd., Dalian, China). Quantitative PCR (qPCR) was performed on a 7500 RealTime PCR system (Applied Biosystems) using a SYBR Green mixing kit (Takara Biotechnology Co., Ltd.) in a 10 μL reaction volume including 1 μL of cDNA. To ensure typical amplification curves and single-peaked melting curves, indicating the presence of a single amplification product for each primer pair (gel electrophoresis can be run to check for single amplification if melting curve analysis is not performed), the coefficient of variation within replicate groups should be equal to or less than 10%, and no signal should be detected in the negative control group. We evaluated mRNA levels using the 2^−ΔΔCT^ method, with *Gapdh* serving as the reference gene. The primer sequences can be found (Additional file 1: Table S2).

### Western blot

Liver and ileum tissues were homogenized and subjected to radio immunoprecipitation assay (RIPA, Beyotime) buffer and phenylmethanesulfonyl fluoride (PMSF, Beyotime) for total protein extraction. The lysate was incubated on ice before being centrifuged at 10,000 × *g* for 15 min. A bicinchoninic acid (BCA) protein assay kit (Beyotime) was used to evaluate the protein concentration. After separation via sodium dodecyl sulfate polyacrylamide gel electrophoresis (SDS-PAGE, Beyotime), the proteins were transferred to polyvinylidene difluoride (MilliporeSigma, Burlington, MA, USA) and blocked with nonfat milk powder in tris buffered saline with Tween-20 (TBST) for 2 h. Next, rabbit monoclonal primary antibodies against Claudin-1, Occludin, zona occludens 1 (ZO-1), transcription factor p65 (p65), p-p65, inhibitor of nuclear factor κB-α (IκB-α), p-IκB-α, TGR5, FXR, cytochrome P4507A1 (CYP7A1), protein kinase A (PKA), p-PKA, cAMP-response element binding protein (CREB), p-CREB, mitogen-activated protein kinase (MEK), p-MEK, extracellular regulated protein kinase (ERK), p-ERK (Cell Signaling Technology, Inc., Danvers, MA, USA), and the rat monoclonal primary antibody against β-actin (Beyotime) were applied to the membranes at a 1:1000 dilution, and the membranes were incubated overnight at 4 °C. After three washes with 1× TBST, the corresponding secondary antibodies, which included horseradish peroxidase (HRP)-labeled goat anti-rabbit immunoglobulin G (IgG) (H + L) and HRP-labeled goat anti-mouse IgG (H + L) (Beyotime) were applied at a 1:1000 dilution for 1 h. Finally, the protein bands were visualized using enhanced chemiluminescence (ECL, Beyotime).

### 16S rRNA

Genomic DNA from cecal intestinal contents was extracted using a fecal DNA kit (Omega Biotek, Inc., Norcross, GA, USA) according to instructions provided by the reagent vendor. Generic primers (341F: 5´-ACTCCTACGGGRSGCAGCAG-3´, 806R: 5´-GGA CTACVVGGGTATCTAATC-3´) were used to amplify the bacterial V3–V4 region of the 16S rRNA gene. The PCR products were then purified using the Qiagen Gel Extraction kit (Qiagen) in order to ensure efficiency and accuracy of amplification. Amplicons were spliced (Flash, version 1.2.11), quality trimmed (Trimmomatic, version 0.33) and chimera removed (Uchime, version 8.1), combined to a uniform concentration and tested on the Illumina HiSeq PE250 platform (Illumina, San Diego, CA, USA) for 2 × 250 bp double-ended read lengths were sequenced. Cluster analysis was performed at a selected 97% similarity level to obtain the corresponding classification units (OTUs). The α-diversity was calculated using QIIME (University of California, San Diego, CA, USA) and β-diversity was assessed by this platform. Linear discriminant analysis and effect size (LEfSe) analyses were performed using the LEfSe tool, and the final relative abundance of bacteria was expressed as a percentage.

### UPLC-MS/MS

Intestinal content samples were separated using a Waters ACQUITY UPLC I-Class system. The mobile phase consisted of phase A, a 0.1% formic acid aqueous solution, and phase B, methanol. Samples were placed in an autosampler at 8 °C, the column temperature was set at 45 °C, flow rate was 300 μL/min, and the injection volume was 2 μL. The liquid phase gradient was as follows: from 0 to 6 min, phase B linearly changed from 60% to 65%; from 6 to 13 min, phase B linearly changed from 65% to 80%; from 13 to 13.5 min, phase B linearly changed from 80% to 90%; from 13.5 to 15 min, phase B remained at 90%. A quality control sample was set at regular intervals in the sample queue to monitor and evaluate system stability and repeatability. Mass spectrometry analysis was performed on a 5500 QTRAP mass spectrometer (AB SCIEX) in negative ion mode. The target ion pairs were detected in multiple reaction monitoring mode. When determining the composition of the contents, mixed standard samples, spiked controls, and test samples were measured in sequence. A standard curve was plotted based on the peak area of the standard samples, and a linear regression equation was calculated. The Multiquant software was used to extract the chromatographic peak area and retention time. Metabolite identification was conducted by correcting the retention time with the bile acid standards.

### Cell culture

Porcine small intestinal epithelial cells (IPEC-J2) and human embryonic kidney cells 293 (HEK293T) were provided by the Animal Nutrition Laboratory of Northeast Agricultural University. IPEC-J2 cells were cultured in DMEM/F-12 complete medium supplemented with 10% fetal bovine serum, streptomycin (100 μg/mL), and penicillin (100 IU/mL). HEK293T cells were cultured in DMEM (4.5 mg/mL glucose) complete medium supplemented with 10% fetal bovine serum, streptomycin (100 μg/mL), and penicillin (100 IU/mL).

### Cell viability

Cells were cultured at a density of 5 × 10^3^ into 96-well microtiter plates, and 100 μL of medium was added to each well until the fusion level reached about 80%. Different concentrations of OA (0, 1, 2, 5, 10, 20, 40, 80, and 100 μmol/L) were added simultaneously to the 96-well microtiter plates to treat the cells for 24 h. The cytotoxicity of OA was determined by using the Cell Counting Kit-8 (CCK-8) (Beyotime) according to the instructions provided by the manufacturer after washing with PBS. Absorbance cells at 450 nm per well were measured using an enzyme marker (Spectra MAX 340, Molecular Devices Co., Sunnyvale, CA, USA). Viability was quantified as a ratio to control.

### Cell treatment

IPEC-J2 cells were seeded at a density of 1 × 10^5^ cells/mL in a 24-well plate. Subsequently, the cells were pre-treated with 0, 5, 10, or 20 μmol/L OA for 12 h and then stimulated with or without 10 μg/mL LPS (*E. coli* O149:B4, Beijing Solarbio Science & Technology Co., Ltd.) for another 6 h. In the correlation assay for pathway inhibition, IPEC-J2 cells were pretreated with siRNA-mate, siRNA TGR5, siRNA FXR, and siRNA-neg (Suzhou GenePharma Co., Ltd., Suzhou, China, 48 h), 20 μmol/L SBI-115 (Guangzhou Bio-gene Technology Co., Ltd., Guangzhou, China, 2 h), 50 μmol/L PD98059 (Guangzhou Bio-gene Technology Co., Ltd., 2 h), 50 μmol/L MDL12330A and 10 μmol/L KH7 (Gamma Scientific Biolab, MA, USA, 2 h), 8 mmol/L H89 and 10 μmol/L PKI (Guangzhou Bio-gene Technology Co., Ltd., 2 h), respectively, and then stimulated with OA and LPS. All cellular experiments had 3 replicates per treatment group, with each experiment repeated 3 times.

### Dual-luciferase reporter assay

A luciferase reporter system was constructed in HEK-293 T cells, which were co-transfected with pCMVSPORT6/hTGR5 and a luciferase reporter plasmid containing the cAMP response element using Lipofectamine 3000 transfection reagent (Thermo Fisher Scientific). Additionally, a human FXR expression vector and a human apical sodium-dependent bile acid transporter (ASBT) expression vector for BA transport were co-transfected into the cells, along with a PGL4-Shp-TK luciferase reporter vector and a Renilla luciferase control vector (pRL-luciferase; Promega, Madison, WI, USA) as a reference. The control luciferase activity was normalized by the Renilla luciferase control vector, and the final data were expressed as the fold induction of luciferase activity compared with the control.

### Statistical analysis

Values are expressed as the mean ± standard deviation of the mean (SD). Statistically significant values were evaluated by one-way analysis of variance (ANOVA) followed by Tukey’s test for multiple comparisons using SPSS v17.0 (SPSS Inc., Chicago, IL, USA). The statistical model included ETEC (Vehicle or ETEC), OA (Control or OA), and their interactions (OA × ETEC) as fixed effects. A threshold of *P* < 0.05 was considered statistically significant.

## Results

### OA improved the diarrhea index and intestinal morphology without affecting growth performance

**Fig. 1:**
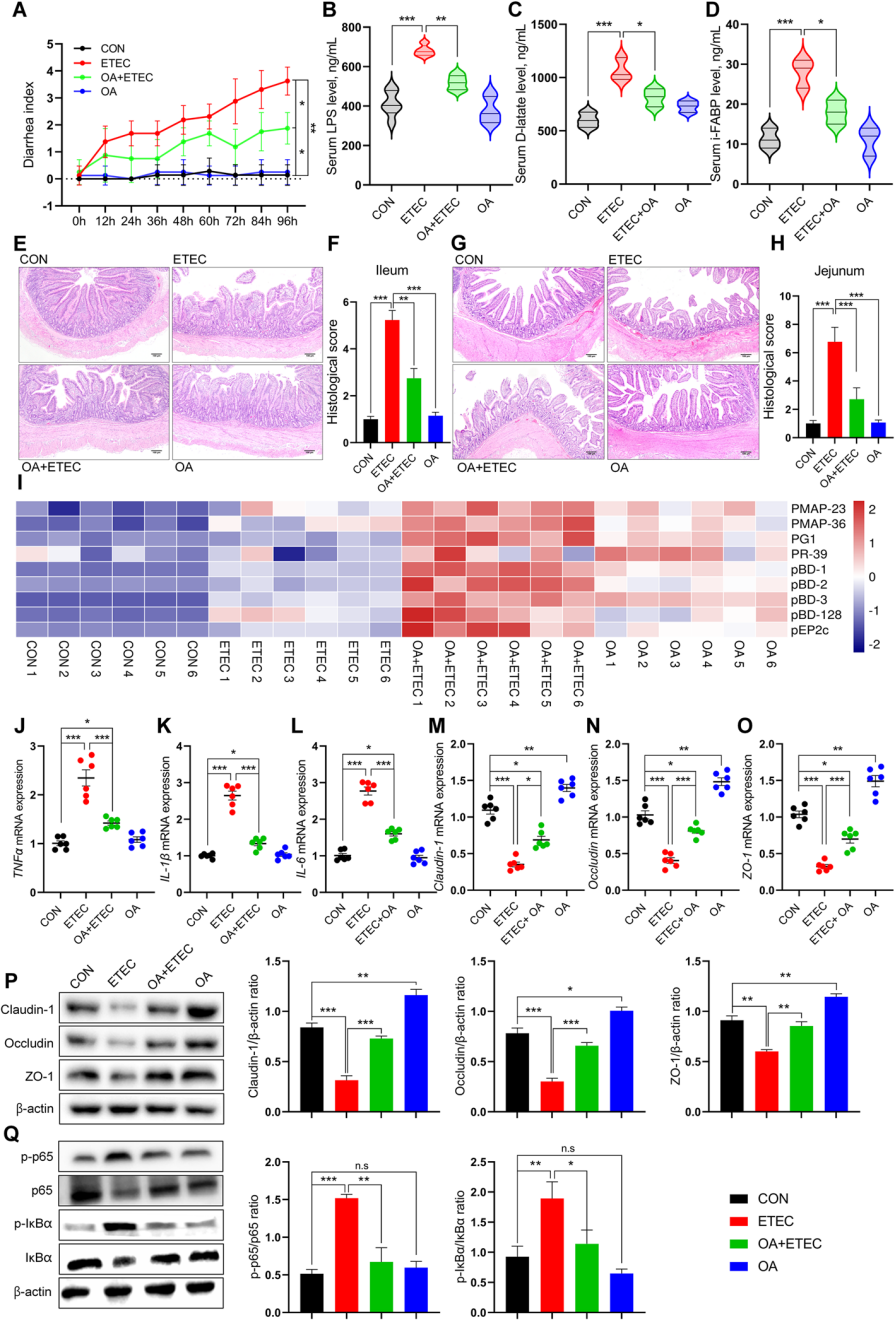
OA alleviates intestinal barrier dysfunction and immune dysregulation caused by ETEC attack. **A** Diarrhea index of piglets in each group after ETEC attack. **B**–**D** Indicators related to intestinal barrier function in serum (LPS, D-latate, i FABP). **E**–**H** Histopathological sections and pathology scoreof jejunum and ileum. **I** Expression of host defense peptides in jejunal mucosa. **J**–**L** Pro-inflammatory factors in the jejunum. **M**–**P** Tight junction protein in jejunum. **Q** NFkB-related protein expression in jejunal tissue. Data are expressed as the mean ± SD and one-way ANOVA was performed, followed by Tukey’s test. ^*^P < 0.05, ^**^P < 0.01, ^***^P < 0.001

We evaluated the effect of OA on growth performance and diarrhea in ETEC-challenged piglets. Throughout the trial, OA supplementation had no significant effect on the average daily gain (ADG) or average daily feed intake (ADFI) (Additional file 1: Table S3). ETEC infection caused a significant reduction in the ADG and ADFI (*P* < 0.05). Dietary OA supplementation did not affect the growth performance of ETEC-challenged piglets. The diarrhea index was used to assess the severity of ETEC-associated diarrhea. Dietary supplementation with OA significantly reduced the diarrhea index in the ETEC-challenged piglets (*P* < 0.05) (Fig. 1A). OA also remediated the morphological damage to the jejunum and ileum caused by ETEC, as evidenced by increased villus length, decreased crypt depth, and pathological index (Fig. 1E–H).

### OA improved barrier function and suppressed ETEC-induced intestinal inflammation

Compared with the control group, ETEC infection significantly increased serum levels of D-lactate, LPS, and iFABP, indicators of an impaired intestinal barrier function. Dietary OA supplementation reduced these levels (Fig. 1B–D). Specifically, OA increased the mRNA and protein abundance of tight junction proteins, including Claudin-1, Occludin, and ZO-1 (Fig. 1N–Q), and induced the expression of host defense peptides (such as pBD, porcine β-defensin; PR-39, Proline-arginine rich 39; PG-1, protegrin-1; PMAP, porcine medullary antimicrobial peptide; pEP2C, porcine epitope protein 2 splice variant C) in the jejunum of ETEC-challenged piglets. Interestingly, OA supplementation also promoted the expression of host defense peptides in the jejunum of uninfected piglets, especially pBD3 (Fig. 1I). OA significantly inhibited the ETEC-induced increase in the transcript expression of jejunal proinflammatory factors (IL-1β, IL-6, and TNF-α) compared with the ETEC group (Fig. 1J–L). Furthermore, OA inhibited ETEC-induced p65 phosphorylation and IκB-α degradation in jejunal tissues (Fig. 1Q). These results indicated that OA enhanced the intestinal barrier and suppressed ETEC-induced intestinal inflammatory responses through the NF-κB signaling pathway.

### OA altered the bile acid and gut microbiota composition

**Fig. 2:**
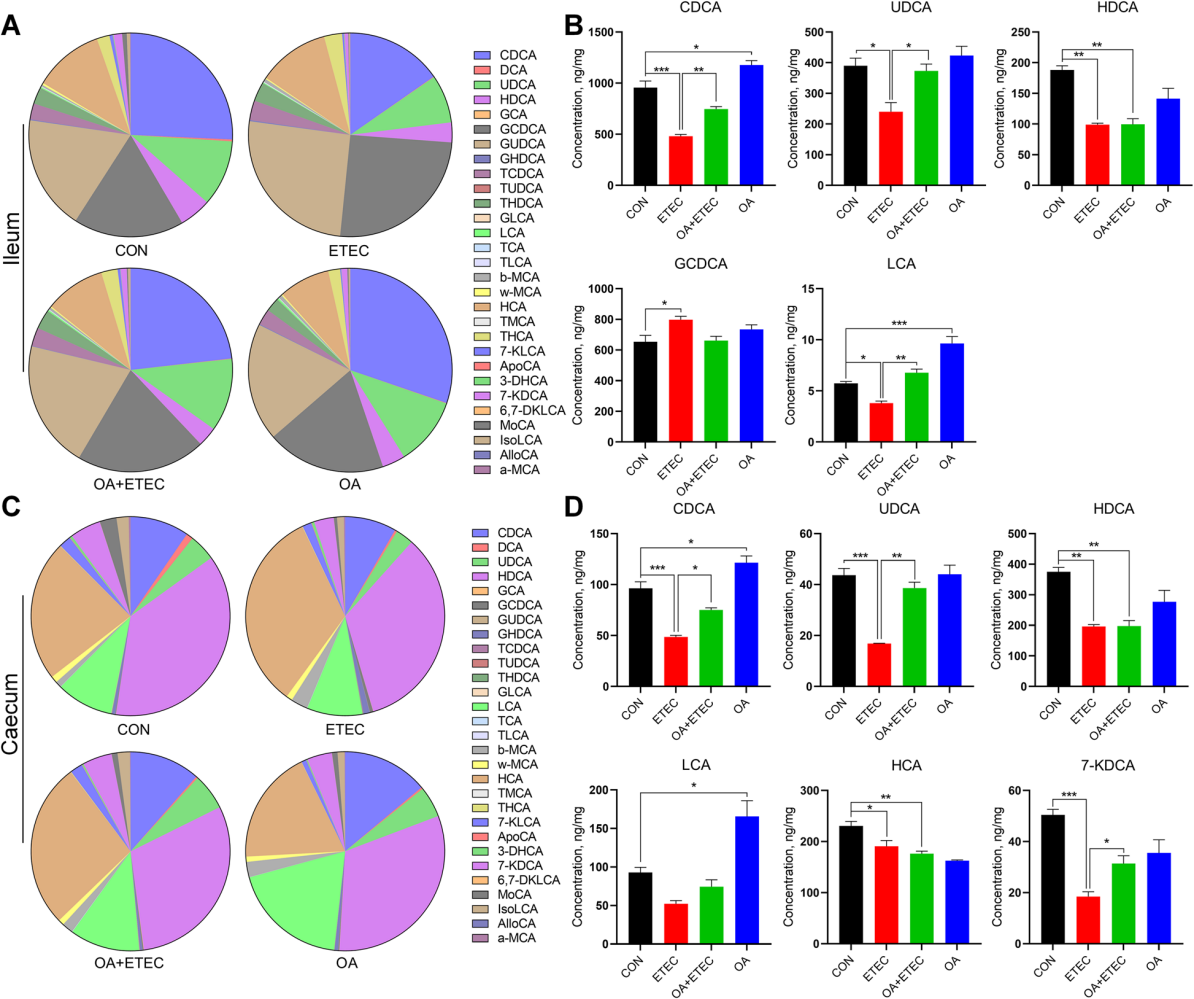
The metabolic profiles of the gut samples from the healthy and ETEC piglets. **A**–**B** Effect of OA on the composition of the ileal bile acid pool. **C**–**D** Effect of OA on the composition of the cecum bile acid pool. Data are expressed as the mean ± SD and one-way ANOVA was performed, followed by Tukey’s test. ^*^P < 0.05, ^**^P < 0.01, ^***^P < 0.001

Gut microbiota dysbiosis plays a critical role in the development of ETEC-induced diarrhea. However, how the gut microbiota mediates the anti-diarrheal effect of OA is unclear. The microbial composition in the cecum was detected by 16S rDNA sequencing. In the ETEC group, the Ace, Chao1, and Shannon indexes decreased, while the Simpson index increased, indicating that the ETEC challenge decreased the richness and diversity of the intestinal microbiota (Additional file 1: Fig. S1A–G). Furthermore, principal component analysis (PCA), principal coordinate analysis (PCoA), and unweighted pair-group method with arithmetic means (UPGMA) revealed a dramatic effect of OA supplementation and ETEC challenge on the gut microbiota structure (Additional file 1: Fig. S1H–J). Specifically, significant differences at the phylum and genus levels were found between piglets in the ETEC and OA + ETEC groups (Additional file 1: Fig. S2A and B). We then used the linear discriminant analysis effect size tool to characterize changes in the gut microbiota and found that *Streptococcus*, *Alloprevotella*, *Campylobacter*, and *Eubacterium coprostanoligenes* were enriched in the ETEC group (Additional file 1: Fig. S2C and D). Lachnospiraceae exhibit bile acid-induced 7α-dehydroxylation activity, converting primary bile acids to secondary bile acids [29]. The total bile acid content in different intestinal tracts was found to be significantly increased in the ileum and colon of piglets due to ETEC (*P* < 0.05, *P* < 0.001), suggesting that ETEC led to over-synthesis of bile acids in the liver and impairment of ileal reabsorption function (Additional file 1: Fig. S3). In contrast, feeding OA significantly reduced the total bile acid content in the ileum and colon of ETEC-attacked piglets and reversed the ETEC-induced damage to the enterohepatic circulation of bile acids in piglets (*P* < 0.05, *P* < 0.01). To investigate whether bile acid components are involved in the process of OA resistance to ETEC-induced diarrhea in piglets, we tested the composition of bile acid pools in the ileum and cecum by LC-MS. There were significant differences in the abundance of primary bile acids between the ileum and cecum in piglets. In the ileal content, the predominant bile acids were chenodeoxycholic acid (CDCA) (25.71%), glycoursodeoxycholic acid (GUDCA) (18.21%), and glycochenodeoxycholic acid (GCDCA) (17.55%). In the cecal content, the main bile acids were hyodeoxycholic acid (HDCA) (37.32%) and hyocholic acid (HCA) (22.94%) (Fig. 2A and C). ETEC infection promotes the formation of GCDCA and GUDCA by binding CDCA and ursodeoxycholic acid (UDCA) to glycine in the ileum. OA significantly increased the levels of UDCA, CDCA, and lithochalic acid (LCA) in both the ileum and cecum of the ETEC-infected piglets (Fig. 2B and D, *P* < 0.05). Compared with those in the control group, the contents of CDCA and LCA in the ileum and cecum were significantly greater in the OA treatment group (Fig. 2B and D, *P* < 0.05).

### OA regulated bile acid metabolism and enterohepatic circulation

**Fig. 3:**
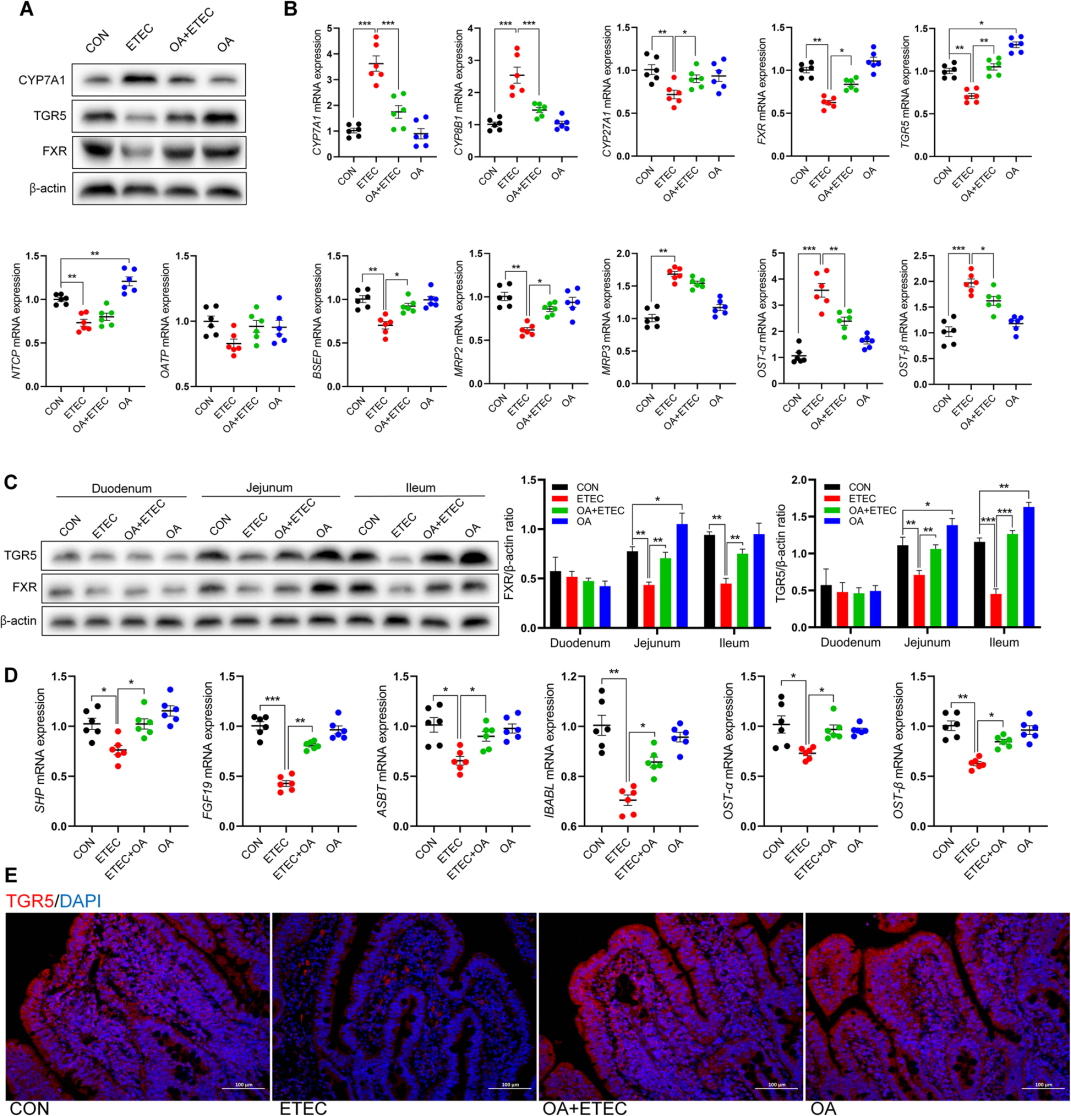
OA improved enterohepatic circulation of bile acids in ETEC-challenged piglets. **A**–**B** Effect of OA on the rate-limiting enzyme for hepatic bile acid synthesis in piglets. **C** Protein expression levels of bile acid receptors in different intestinal segments. **D** Effect of OA on mRNA expression levels of genes required for ileal bile acid transport in piglets. **E** Immunohistofluorescence of TGR5 in jejunum. ASBT, apical sodium-dependent bile acid transporter; BSEP, bile salt export pump; CYP27A1, cytochrome P45027A1; CYP7A1, cytochrome P4507A1; CYP8B1, cytochrome P4508B1; FGF19, fibroblast growth factor 19; FXR, farnesoid X receptor; iFABP, intestinal-type fatty acid binding protein; MRP2, multidrug resistance-associated protein 2; MRP3, multidrug resistance-associated protein 3; NTCP, Na^+^-taurocholic acid cotransport protein; OATP, organic anion transporting polypeptides; OST-α/β, organosolute α/β heterodimer; TGR5, takeda G protein-coupled receptor 5; SHP, small heterodimer partner. Data are expressed as the mean ± SD and one-way ANOVA was performed, followed by Tukey’s test. ^*^P < 0.05, ^**^P < 0.01, ^***^P < 0.001

We measured the expression of rate-limiting enzymes and bile acid receptors in the bile acid synthesis pathway to clarify the regulatory mechanism of OA in the bile acid pool. In the liver, ETEC upregulated the mRNA expression of cytochrome P4507A1 (*CYP7A1*) and cytochrome P4508B1 (*CYP8B1*) in the classical bile acid synthesis pathway and downregulated the mRNA expression of cytochrome P45027A1 (*CYP27A1*) in the alternative synthesis pathway and the bile acid receptors FXR and TGR5 [30] (Fig. 3B). OA supplementation reversed the trend of mRNA changes in ETEC bile acid synthesis rate-limiting enzymes and bile acid receptors, and significantly increased the mRNA expression of *TGR5* (*P* < 0.05). Western blot analysis verified the regulatory effects of OA on CYP7A1, FXR, and TGR5 in the liver of ETEC-challenged piglets (Fig. 3A). ETEC also significantly inhibited the mRNA expression of Na^+^-taurocholic acid cotransport protein (*NTCP*) in the liver (*P* < 0.001), thereby inducing de novo bile acid synthesis [31]. At the same time, ETEC significantly inhibited the mRNA expression of bile salt export pump (*BSEP*) and multidrug resistance-associated protein 2 (*MRP2*) (*P* < 0.001), suggesting that the delivery of liver bile acids to the gallbladder was impaired [32]. ETEC also increased the mRNA expression of *MRP3*, *OST-α*, and *OST-β* in the liver of piglets (*P* < 0.001), leading to the excretion of bile acids in the systemic circulation (Fig. 3B) [33]. OA supplementation reversed these ETEC-induced disturbances in hepatic bile acid storage and transport.

In the small intestine, OA significantly increased the abundance of bile acid receptors FXR and TGR5 in the jejunum and ileum epithelium (Fig. 3C). Immunofluorescence confirmed the OA-driven overexpression of TGR5 in the jejunum (Fig. 3E). The expression of genes related to bile acid transport in the piglet ileum was also detected. ETEC significantly suppressed the mRNA expression of the FXR downstream genes small heterodimer partner (*SHP*) and fibroblast growth factor 19 (*FGF19*), and unsurprisingly, reversed by OA. ETEC also inhibited the mRNA expression of bile acid binding proteins and transporters, such as *ASBT*, *OST-α*, *OST-β*, and ileal bile acid-binding protein (*IBABP*) (*P* < 0.01), which would reduce the reabsorption of bile acids transported by enterocytes into the portal vein, thereby disrupting the enterohepatic circulation of bile acids [34]. However, OA inhibited the re-synthesis of bile acids in the liver by increasing the expression of the FXR signaling pathway and negative feedback, while increasing the expression level of bile acid transporters, thereby regulating the homeostasis of bile acid circulation (Fig. 3D).

### OA alleviated LPS-induced intestinal epithelial cell inflammation through the TGR5-cAMP-PKA-NF-κB pathway

**Fig. 4:**
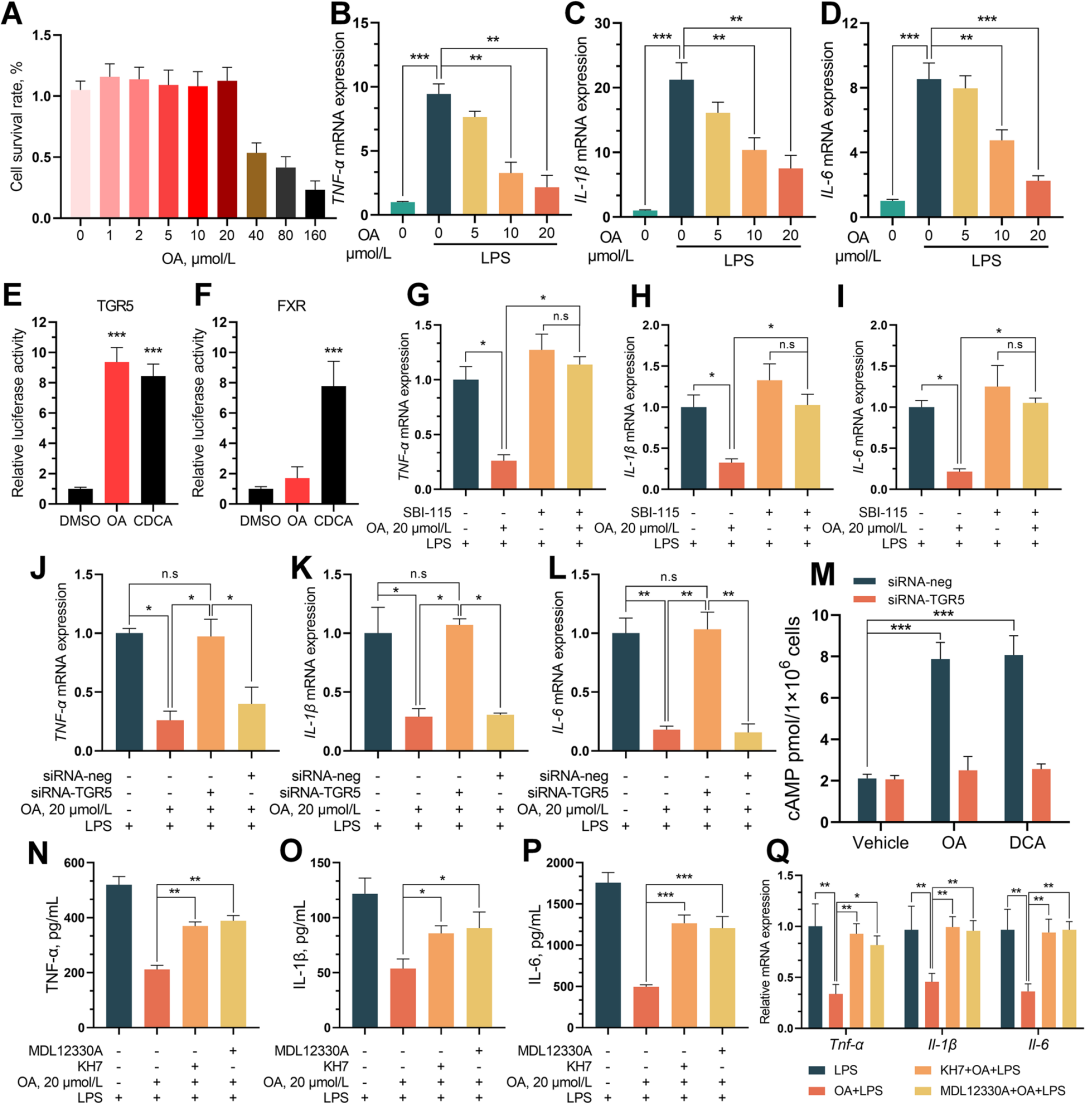
OA alleviates LPS-induced inflammation in IPEC-J2 by activating TGR5. **A** Effect of different concentrations of OA on the viability of IPEC-J2 cells. **B**–**D** OA pretreatment inhibited LPS-stimulated pro-inflammatory factor overexpression in IPEC-J2 cells. **E **and** F** Dual-luciferase reporter assay for TGR5 and FXR. **G**–**I** SBI-115 pretreatment eliminated the inhibition of LPS-induced inflammation by OA. **J**–**L** Effect of TGR5 signal silencing on anti-inflammatory function of OA. **M** Detection of intracellular cAMP levels in IPEC-J2 cells under different treatments. **N**–**Q** The adenylate cyclase-specific inhibitors KH7 and MDL12330A blocked the effects of OA on LPS-stimulated proinflammatory factor expression. Data are expressed as the mean ± SD and one-way ANOVA was performed, followed by Tukey’s test. ^*^P < 0.05, ^**^P < 0.01, ^***^P < 0.001

To explore the molecular mechanism of OA-mediated inhibition of ETEC-induced intestinal inflammation in piglets, we established a model of intestinal epithelial cell inflammation by stimulating IPEC-J2 cells with LPS (10 μg/mL). Cytotoxicity was measured with a cell counting kit-8 (CCK-8) assay, which indicated that the maximum nontoxic concentration of OA was 20 μmol/L, and subsequent studies were conducted within this concentration range (Fig. 4A). Consistent with the in vivo results, OA inhibited the LPS-induced inflammatory response in IPEC-J2 cells in a dose-dependent manner (Fig. 4B–D). Given that OA induces FXR and TGR5 expression in the piglet jejunum, we examined whether OA activates bile acid receptors in intestinal epithelial cells (IECs). Interestingly, OA strongly inhibited TGR5 but did not affect FXR according to the results of cell-based reporter assays (Fig. 4E and F). To confirm the role of TGR5 activation in the anti-inflammatory effects of OA, we blocked TGR5 activation with the specific inhibitors SBI-115 or siRNA-TGR5 [35]. Both SBI-115 and siRNA-TGR5 reversed the protective effect of OA on the production of LPS-induced proinflammatory cytokines (IL-1β, IL-6, and TNF-α) in IPEC-J2 cells (Fig. 4G and L).

**Fig. 5:**
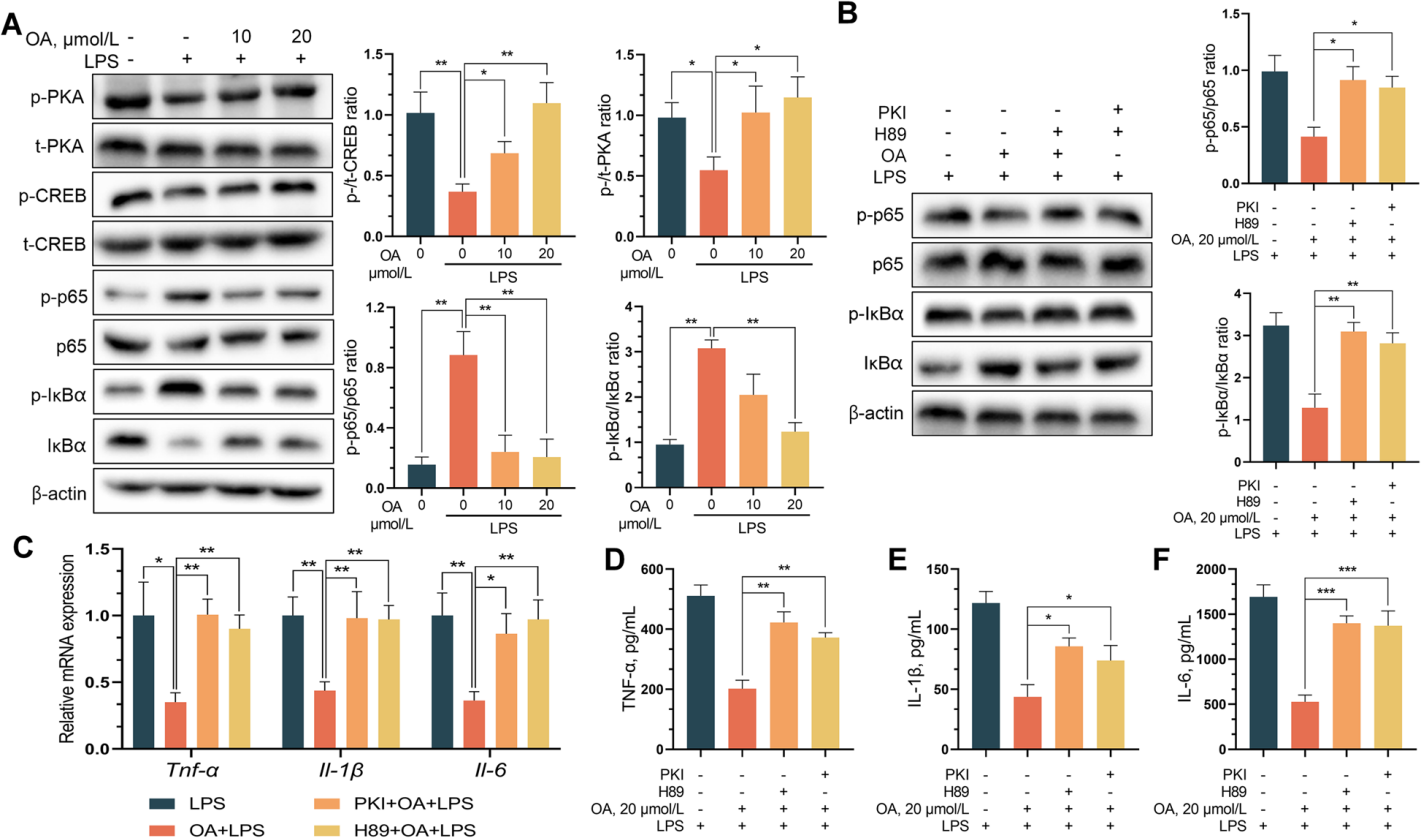
OA alleviates ETEC-induced inflammation in IPEC-J2 through the cAMP-PKA- NF-κB pathways. **A** The protein levels of cAMP-PKA- NF-κB pathways from the indicated groups were determined by western blotting. **B** The PKA-specific inhibitors H89 and PKI eliminated the inhibitory effect of OA on NF-κB-mediated inflammatory factor overexpression. **C **and** D** The relative mRNA and protein levels of proinflammatory factors from the indicated group were detected by qPCR. Data are expressed as the mean ± SD and one-way ANOVA was performed, followed by Tukey’s test. ^*^P < 0.05, ^**^P < 0.01, ^***^P < 0.001

Previous studies have shown that ligand binding to TGR5 contributes to the production of cAMP, which leads to PKA activation. We examined the effect of OA on intracellular cAMP levels in IPEC-J2 cells and found that similar to deoxycholic acid (DCA), OA significantly increased the intracellular cAMP concentration in IPEC-J2 cells. TGR5 blockade abolished cAMP induction by OA (Fig. 4M). We verified the cAMP-mediated anti-inflammatory effect of OA using the adenylyl cyclase inhibitors MDL12330A and KH7 and found that both reversed the OA-induced decrease in inflammatory cytokine expression in LPS-treated IPEC-J2 cells (Fig. 4 N–Q) [36]. cAMP inhibits the transcriptional activity of NF-κB and reduces the transcription of inflammatory factors by activating the PKA/CREB signaling pathway. Western blot analysis showed that OA promoted the phosphorylation of PKA and CREB in IPEC-J2 cells in a dose-dependent manner. Unexpectedly, OA suppressed LPS-induced P65 phosphorylation and IKB degradation (Fig. 5A). Pretreatment with the PKA-specific inhibitors PKI and H89 abolished the inhibitory effect of OA on LPS-induced inflammation (Fig. 5A) [37]. Pretreatment with the PKA-specific inhibitors PKI and H89 abrogated the ability of OA to suppress NF-κB nuclear translocation (Fig. 5B) and impaired the ability of OA to suppress LPS-induced inflammation (Fig. 5C–F). These data suggest that OA inhibits the LPS-induced nuclear translocation of NF-κB-p65 by activating the cAMP/PKA/CREB signaling pathway.

### OA enhanced CDCA-activated FXR signaling and induced pBD3 expression in IECs through MEK/ERK signaling

**Fig. 6:**
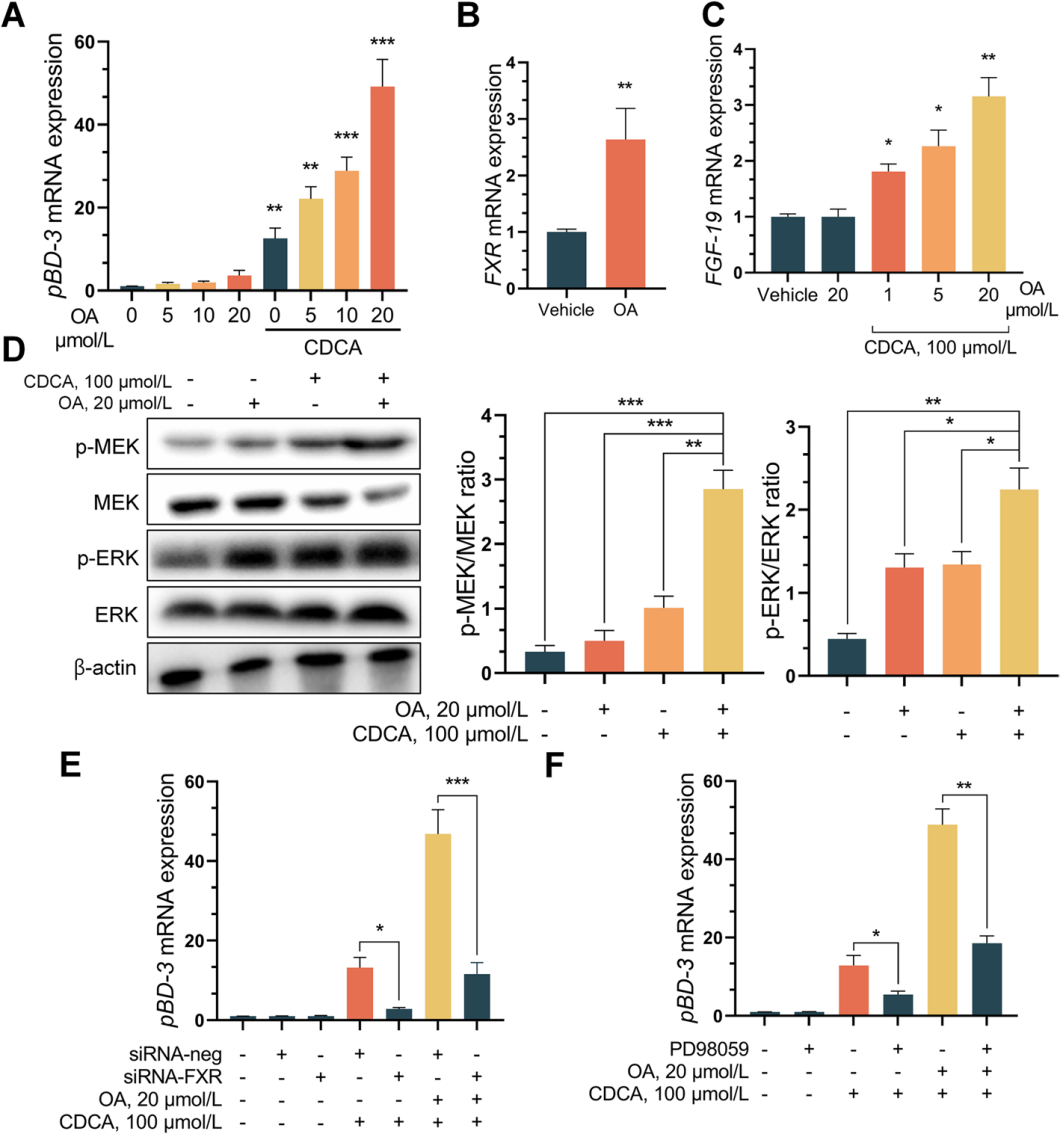
OA and CDCA co-stimulate the expression of pBD3 in IPEC-J2. **A** OA and CDCA co-stimulated the expression of pBD3 in IPEC-J2. **B** OA induced the mRNA expression of *FXR* in IPEC-J2. **C** *FGF-19* mRNA expression were analyzed by qRT-PCR. **D** Expression detection of proteins related to MEK-ERK signaling pathway. **E **and** F** Effect of silencing or inhibiting FXR and MEK on OA and CDCA-stimulated pBD3 expression. Data are expressed as the mean ± SD and one-way ANOVA was performed, followed by Tukey’s test. ^*^P < 0.05, ^**^P < 0.01, ^***^P < 0.001

Supplementing OA increased the expression of host defense peptides, especially pBD3, in the jejunum of piglets (Fig. 1I). However, the effect of stimulating pBD3 by OA in IPEC-J2 cells in vitro was unlike that in vivo. Considering that OA supplementation significantly increased the content of the primary bile acid CDCA in the lumen of the small intestine, we examined the effect of CDCA on the expression of pBD3 in IPEC-J2 cells. Interestingly, as the OA concentration increased, the effect of CDCA on stimulating the expression of pBD3 was enhanced (Fig. 6A). Given that CDCA is a natural ligand of FXR, we sought to investigate whether the regulatory effect of OA on the FXR expression was involved in the activation of FXR by CDCA. Similar to our findings in piglets, OA significantly upregulated the expression of *FXR* mRNA (Fig. 6B). Furthermore, OA pretreatment significantly increased CDCA-induced mRNA expression of the FXR target gene *FGF-19* in comparison with CDCA alone (Fig. 6C). Previous studies have shown that the MEK-ERK signaling pathway plays an important role in the expression of intestinal host defense peptides [38]. Therefore, the effect of OA preconditioning on the activation of the MEK/ERK pathway by CDCA was analyzed. OA significantly enhanced the induction of MEK and ERK phosphorylation by CDCA (Fig. 6D). After blocking FXR with siRNA or inhibiting MEK with PD98059, OA failed to cooperate with CDCA to induce pBD3 expression in IPEC-J2 cells (Fig. 6E and F). These results demonstrated that OA amplifies FXR signaling and stimulates CDCA to induce pBD3 expression in IECs via MEK-ERK.

## Discussion

Diarrhea is a common health issue in young children and animals, particularly during the early stages of life. This is because their intestinal and immune functions are not yet fully understood, increasing susceptibility to infections and other illnesses [39]. ETEC is a common cause of diarrhea in juvenile animals, including calves, piglets, and lambs. Young animals infected with ETEC often die due to severe watery diarrhea and rapid dehydration, resulting in immediate financial loss to farmers [40]. After entering the intestine, ETEC can use bacterial surface adhesins to adhere to the IECs and secrete enterotoxins, causing intestinal inflammation and injury [41] and even endotoxemia or systemic infection [42]. Since the prohibition of antibiotics in livestock production, there has been a lack of effective nutritional measures available to mitigate ETEC infections. OA is a representative compound of pentacyclic triterpenoids found in edible or medicinal plants. We previously reported the protective effects of OA on the intestinal barrier and immune homeostasis in rodent models, suggesting that OA could be used as a novel foodborne therapy for intestinal diseases caused by pathogenic bacterial infections [18, 19]. In the present study, dietary OA supplementation was effective at preventing diarrhea and intestinal damage caused by ETEC infection in piglets. OA manifests as protection of the intestinal mucosal structure and barrier function, control of the inflammatory response, enhancement of the antimicrobial response, and improvement of the microbiota. In healthy piglets not infected with ETEC, OA supplementation had little effect on the structure of the gut microbiota, suggesting that OA may indirectly balance the disturbed gut microbiota in ETEC-infected piglets rather than act directly on the microorganisms (Fig. 1 and Additional file 1). Therefore, we conducted an in-depth study of the “indirect” mechanism of OA function.

Bile acids are a class of organic acids with a steroidal structure that are synthesized from cholesterol by liver cells, stored in the gallbladder, and continuously circulated between the intestines and the liver [43]. Specifically, cholesterol is directly converted to primary free bile acids in hepatocytes. Primary free bile acids are combined with taurine or glycine, respectively, to form primary conjugated bile acids in the liver, which are secreted through the bile ducts to the gallbladder for storage and excreted into the duodenum with food. About 90% of the primary conjugated bile acids return to the liver via active reabsorption at the end of the ileum, and a small portion is reduced to primary free bile acids by de-conjugation of the intestinal microbiota, and secondary free bile acids are formed by dehydroxylation of the intestinal microbiota. Primary and secondary free bile acids return to the liver via passive reabsorption in the anterior small intestine and colon. The free bile acids that return to the liver are converted to conjugated bile acids, which, together with the reabsorbed and newly synthesized conjugated bile acids, are excreted into the intestine with bile, thus forming the enterohepatic cycle of bile acids [44]. In recent years, extensive research has shown that bile acids possess multiple physiological functions. They not only participate in the metabolism of lipid substances in the body [45], but also exhibit a protective effect on intestinal health [46, 47]. Bile acids, within the process of enterohepatic circulation, can inhibit pathogenic bacteria in the intestine and maintain the intestinal epithelial barrier to prevent intestinal diseases [48]. Additionally, they regulate intestinal immune function and maintain immune tolerance to harmless antigens in the gut [49]. The development of inflammatory bowel disease (IBD) has been found to be associated with the disruption of the intestinal bile acid pool. Cohort studies, including the Integrated Human Microbiome Project, have shown that the pathogenesis of IBD is accompanied by a decrease in secondary bile acids [50]. Supplementation with secondary bile acids ameliorates the pathologic features of intestinal inflammation in acute and chronic mouse models of colitis [51]. Utilizing these potential bile acid molecules can provide new avenues for treating intestinal diseases. In this study, intestinal flora dysbiosis caused by ETEC infection resulted in altered bile acid composition in the ileum and cecum of piglets. In particular, ETEC increased the levels of GCDCA and GUDCA (composed of CDCA and UDCA combined with glycine, respectively) in the ileum of piglets (Fig. 2B). OA feed supplementation can significantly increase the contents of CDCA, UDCA, and LCA in the ileum and cecum of ETEC-challenged piglets (Fig. 2B and D). GUDCA, an endogenous antagonist of the bile acid receptor FXR, can selectively antagonize the intestinal FXR signaling pathway to affect bile acid metabolism [52]. This suggests that ETEC may affect the conversion of UDCA to GUDCA and thus inhibit ileal FXR expression, resulting in the disruption of bile acid metabolism (Fig. 2B and D). UDCA and its primary metabolite LCA inhibit epithelial cell apoptosis, promote the intestinal barrier function, and reduce the production of proinflammatory cytokines. A mixture of LCA and UDCA has been shown to upregulate the expression of tight junction proteins, thereby repairing intestinal barrier integrity. Studies on early-weaned piglets also showed that CDCA improved intestinal mucosal morphology and upregulated the expression of the tight junction protein ZO-1 in piglets [53]. In addition, CDCA and LCA are good endogenous agonists of the bile acid receptors FXR and TGR5, respectively. Sorribas et al. [54] found that agonists of FXR exert beneficial effects on intestinal inflammation and the intestinal barrier. Sorrentino et al. demonstrated that TGR5 activation mediates intestinal cell regeneration in the intestine and ameliorates intestinal epithelial damage in a mouse model of colitis [55]. This evidence suggests that OA may protect piglets from ETEC-induced intestinal injury and inflammation by modulating bile acid composition in the intestine.

The enterohepatic circulation of bile acids is mediated by several binding and transport proteins. Along with MRP2, bile acids enter the intestine via the BSEP, where they are received by the ASBT in the ileum and reach the intestinal epithelium. Subsequently, bile acids bind to IBABP and are transported to the basement membrane via organ solute α/β heterodimers (OST-α/β) for export to the portal vein. Upon return to liver tissue, bound bile acids are recovered by NTCP, and free bile acids are transported by organic anion-transporting polypeptides [56]. The dynamic balance of the enterohepatic circulation of bile acids maintains homeostatic bile acid metabolism; once this balance is disrupted, bile acid metabolism becomes impaired, promoting the development of intestinal and hepatic diseases, which in turn trigger pathological changes and metabolic disorders of the intestine and liver [57]. The most typical example is bile acid diarrhea. Patients exhibit impaired reabsorption of bile acids in the ileum, leading to a significant influx of bile acids from the ileum into the colon. This results in elevated concentrations of bile acids in the colon, exceeding normal physiological levels. Simultaneously, it induces colonic epithelial cells to secrete Cl^−^, enhancing bowel movements and ultimately causing the patient to experience secretory diarrhea [58]. In this study, ETEC caused a significant increase in total bile acids in the ileum and colon of piglets, which suggests that ETEC leads to over-synthesis of bile acids in the liver and impairment of ileal reabsorption. In contrast, feeding OA significantly reduced the total bile acid content in the ileum and colon of ETEC-attacked piglets and reversed the ETEC-induced damage to the enterohepatic circulation of bile acids in piglets. Furthermore, we found that ETEC infection significantly inhibited the expression of the ileal bile acid receptor FXR as well as downstream SHP and FGF19, indicating that ETEC inhibited the negative feedback regulation of the FXR signaling pathway on hepatic bile acid synthesis (Fig. 3D) [59]. ETEC also inhibited the mRNA expression of the ileal bile acid transport-related proteins *ASBT*, *IBABP*, *OST-α*, and *OST-β*, which further inhibited the uptake and transport of intestinal bile acids by intestinal epithelial cells, thereby disrupting the dynamic balance of the enterohepatic circulation of bile acids (Fig. 3D) [60]. In addition, ETEC inhibited the expression of NTCP (responsible for the recovery of bound bile acids [61]) and increased the expression of CYP7A1 and CYP8B1, the rate-limiting enzymes of the classical bile acid synthesis pathway [62]. This inhibited hepatic uptake of bile acids from the portal vein and induced further bile acid resynthesis in the liver (Fig. 3B). ETEC also reduced the levels of BSEP and MRP2 in the liver, which attenuated the transport of bile acids to the gallbladder [63], ultimately leading to disturbances in bile acid metabolism (Fig. 3B). OA supplementation negatively regulates the hepatic synthesis of bile acids by increasing the expression levels of the bile acid receptors FXR and TGR5 and their target genes *SHP* and *FGF19* in the liver and ileum. Moreover, OA partially reversed the detrimental changes in bile acid metabolism enzymes and transport proteins in the ileum and liver caused by ETEC (Fig. 3B and D). These findings demonstrated that OA regulates the uptake, transport, and delivery of bile acids to the intestine and liver, which maintains the dynamic balance of the enterohepatic circulation of bile acids and ultimately ensures the normal homeostasis of bile acid metabolism.

The cAMP-PKA-CREB signaling pathway is mediated by the bile acid receptor TGR5 and is involved mainly in the body’s anti-inflammatory immune regulation process [64]. In APP/PS1 transgenic mice, activation of cAMP-PKA-CREB signaling inhibits the expression of NF-κB, thereby exerting an anti-inflammatory effect. Specifically, activated by cAMP, PKA can phosphorylate the inhibitor of kappa B kinase (IKK) complex, specifically the regulatory subunit IKKβ. This phosphorylation event can negatively regulate the IKK complex, thereby preventing the phosphorylation and degradation of IκBα [65]. As a result, NF-κB remains sequestered in the cytoplasm, and its transcriptional activity is suppressed. In addition, the CREB protein can physically interact with the p65 subunit of NF-κB, sequestering it in the cytoplasm and preventing its nuclear translocation. This interaction inhibits the ability of NF-κB to promote the transcription of proinflammatory target genes [66]. Pentacyclic triterpenoids, a group of naturally occurring compounds widely found in plants, have attracted significant amounts of attention due to their diverse pharmacological properties, including anti-inflammatory, antioxidant, and anti-tumor activities. Among these triterpenoids, oleanolic acid has emerged as a selective TGR5 agonist (EC_50_ 2.25 μmol/L) [67]. Upon activation by oleanolic acid or other ligands, TGR5 initiates a signaling cascade involving the production of cAMP, which in turn activates PKA and downstream transcription factors, such as CREB [68]. Therefore, we studied whether OA mediates the cAMP-PKA-CREB signal transduction pathway through the TGR5 receptor to explore the molecular mechanism through which OA is independent of bile acid. The results showed that OA could significantly increase cAMP levels and the expression and activity of PKA and CREB in IPEC-J2 cells (Fig. 4M and 5A). The adenylate cyclase-specific inhibitors KH7 and MDL12330A blocked OA’s inhibition of LPS-stimulated proinflammatory factor overexpression, indicating that OA inhibited inflammatory signaling in a CAMP-dependent manner after activating TGR5 (Fig. 4N–Q). Furthermore, we verified the critical role of the PKA-CREB signaling pathway in the OA-mediated inhibition of the inflammatory response in intestinal epithelial cells. The results revealed that the PKA-specific inhibitors H89 and PKI eliminated the inhibitory effect of OA on NF-κB-mediated inflammatory factor overexpression in intestinal epithelial cells (Fig. 5B–F). This evidence fully demonstrates that OA exerts its inhibitory effect on intestinal inflammation by directly activating TGR5 and initiating the cAMP-PKA-CREB signaling pathway.

Host defense peptides (HDPs) are a group of genetically encoded cationic small peptides that are essential effector molecules of the innate immune system. In the intestine, the high expression of host defense peptides evidences the enhancement of the immune function in the organism [69, 70]. In this study, OA supplementation can significantly increase the expression of host defense peptides in the jejunum of piglets. Notably, the activation of TGR5 by OA cannot explain the role of OA in inducing the expression of intestinal defense peptides in piglets. Interestingly, OA treatment did not significantly induce the expression of defense peptides in IPEC-J2 cells, contrary to the in vivo results. Therefore, we considered whether bile acids are involved in the induction of defense peptide production by OA and found that CDCA and OA costimulated the expression of pBD3 in IPEC-J2 cells. CDCA has been demonstrated to induce antimicrobial programs in various types of intestinal epithelial cells, including paneth cells, goblet cells, and enterocytes [68]. Considering these findings and the fact that the primary unconjugated bile acid CDCA does not require active transport to enter intestinal epithelial cells, CDCA can interact with the nuclear receptor FXR once it is passively taken up by cells [71]. Although OA has no direct activating effect on FXR, CDCA is a natural agonist of FXR; therefore, we hypothesized that OA regulates FXR to produce a synergistic effect with CDCA. Interestingly, our research findings indicate that although OA is unable to directly activate FXR signaling, it can modulate the intensity of CDCA-mediated FXR signaling by increasing FXR expression in intestinal epithelial cells (Fig. 4F and 6B). Treating IPEC-J2 cells with OA increased FXR expression in a concentration and time-dependent manner. Moreover, this enhanced receptor expression was associated with an amplified CDCA-mediated antimicrobial response in IPEC-J2 cells (Fig. 6A). As expected, treatment with OA alone increased FXR activity in intestinal epithelial cells, as evidenced by the elevated expression of FGF-19 (Fig. 6C). In general, differential induction of pBD3 in IPEC-J2 cells after stimulation with CDCA was observed (Fig. 6A). Recently, in a third type of epithelial cell, namely, cholangiocytes, primary bile acids were shown to increase VDR expression through the MEK-ERK signaling pathway, leading to elevated levels of cathelicidin [72]. Consequently, primary bile acids such as chenodeoxycholic acid and ursodeoxycholic acid increase cathelicidin levels either alone or in the presence of 1,25D3 (the active ligand for VDR) [72]. CDCA, a potent agonist of FXR, also has a regulatory effect on the MEK-ERK signaling pathway [73]. The mechanism of CDCA treatment in liver cancer is associated with the upregulation of CYP1A1 induced by the activation of MEK1/2 [73]. FXR first activates GTPase and RAS, followed by the activation of RAF kinase at the plasma membrane. Activated RAF kinase phosphorylates the downstream MEK, promoting RAF-mediated activation of MEK. Activated MEK then doubly phosphorylates ERK, which can translocate to the cell nucleus and activate various transcription factors [74, 75]. ERK is involved in the proper function of antimicrobial programs in host cells [76, 77] and, in particular, plays a crucial role in the regulation of pBD-3 [78]. Nutrients such as short-chain fatty acids and isoleucine have been reported to stimulate the expression of host defense peptides through the MEK-ERK signaling pathway [79]. Similarly, increased levels of hBD-2 in the serum of psoriasis patients are due to inflammation promoting the activity of the immune system, which in turn elevates the phosphorylation of JNK, ERK, and Akt, subsequently enhancing hBD-2 expression [79]. Examination of the phosphorylation levels of the relevant proteins revealed that OA synergistically activated the FXR-MEK-ERK signaling pathway with CDCA and induced the expression of *pBD3* mRNA in intestinal epithelial cells (Fig. 6D). After blocking FXR with siRNA or inhibiting MEK with PD98059, the tendency of OA and CDCA to synergistically promote pBD3 expression was suppressed (Fig. 6E and F). Overall, we propose that OA enhances FXR signaling in intestinal epithelial cells, thereby synergistically activating the MEK-ERK signaling pathway through CDCA to induce the expression of defensive factors.

## Conclusions

**Fig. 7:**
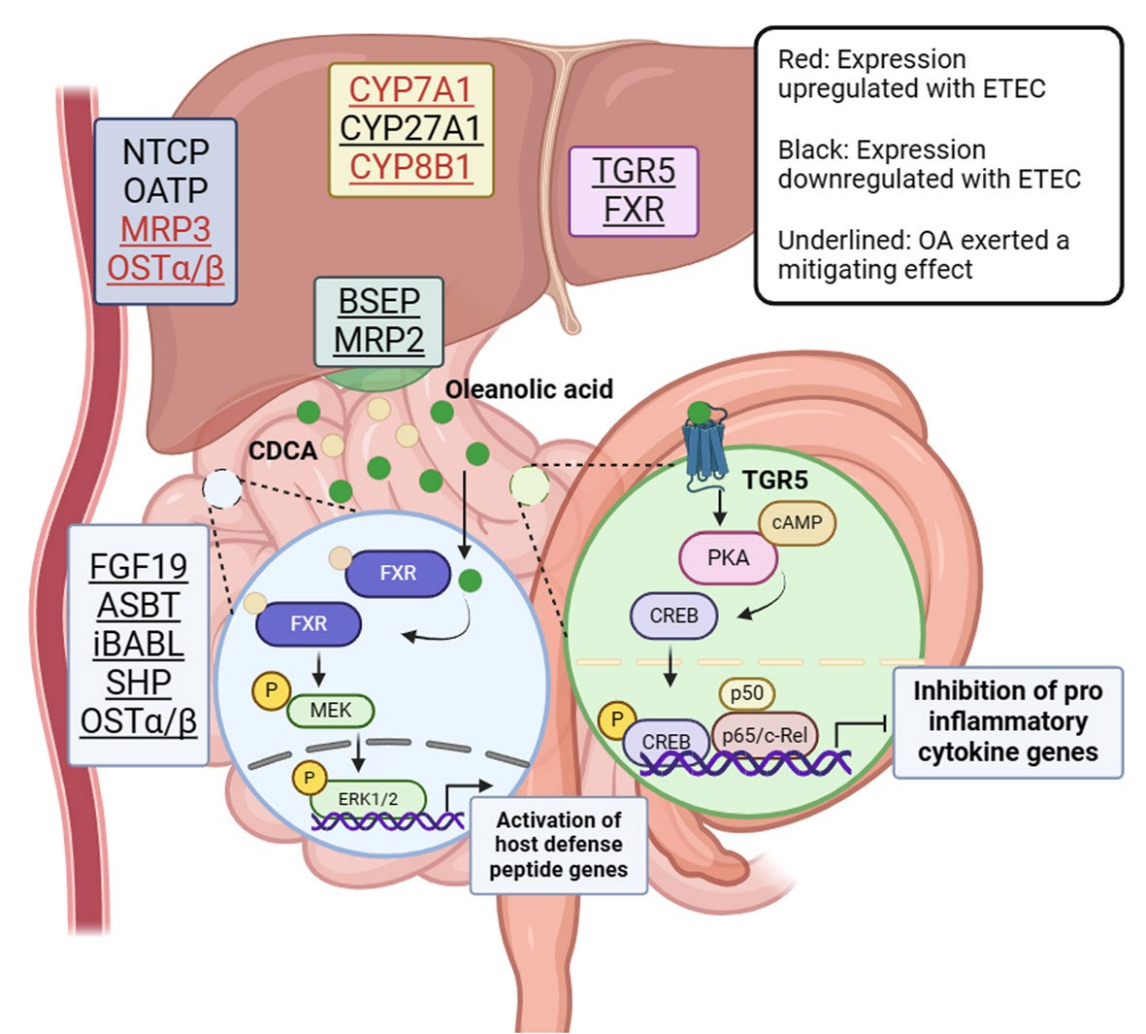
OA improved the bile acid enterohepatic circulation in ETEC-challenged piglets and maintained intestinal immune homeostasis by activating and enhancing bile acid receptor signaling

In summary, our results demonstrate that compared to healthy piglets, ETEC-infected piglets exhibited disrupted bile acid metabolism. ETEC infection promoted the formation of GCDCA and GUDCA by binding CDCA and UDCA with glycine in the ileal contents. Feeding OA improved the enterohepatic circulation of bile acids, affecting the composition of the piglet bile acid pool, and increasing the levels of UDCA and CDCA in the ileum and cecum. OA’s regulatory effect on bile acid metabolic homeostasis not only suppressed ETEC-induced intestinal inflammation in piglets but also enhanced the intensity of the piglet’s intestinal antimicrobial programs. The underlying mechanism is related to TGR5 and FXR. Specifically, OA inhibited NF-κB-mediated intestinal inflammation by directly activating TGR5 and its downstream cAMP-PKA-CREB signaling pathway. Additionally, OA enhanced CDCA-mediated MEK-ERK signaling by up-regulating FXR expression in intestinal epithelial cells, thereby activating anti-microbial responses in the intestine (Fig. 7). These findings suggest that disruption of bile acid enterohepatic circulation and biota-mediated secondary acid production are key regulators in the pathogenesis of ETEC-associated enteritis, and that OA may act as a functional food or feed additive to regulate intestinal innate immunity through the “bile acid-TGR5/FXR” axis.

### Supplementary Information

**Additional file 1: Table S1** Ingredients and compositions of the basal diet; **Table S2** Sequences of primers used for real-time polymerase chain reaction; **Table S3** Effect of OA on productive performance of ETEC-infected piglets; **Fig. S1** Effects of OA on the diversity, richness, and structure of gut microbiota in ETEC-challenged piglets; **Fig. S2** Compositions of cecum microbiota at the phylum (**A**) and genus (**B**) level. **C** and **D** The linear discriminant analysis (LDA) effect size was calculated to explore the taxa within genus levels that more strongly discriminated between the gut microbiota of mice fed with ETEC compared to CON group or ETEC+OA compared to ETEC; **Fig. S3** Total bile acids in the contents of the ileum and colon of piglets.

## Data Availability

The original contributions presented in the study are included in the article/ Supplementary Material, and further inquiries can be directed to the corresponding author.

## Abbreviations

ADFI: Average daily feed intake
ADG: Average daily gain
ASBT: Apical sodium-dependent bile acid transporter
BCA: Bicinchoninic acid
BSEP: Bile salt export pump
CAR: Constitutive androstane receptor
CCK-8: Cell counting kit-8
CDCA: Chenodeoxycholic acid
CREB: cAMP-response element binding protein
CYP27A1: Cytochrome P45027A1
CYP7A1: Cytochrome P4507A1
CYP8B1: Cytochrome P4508B1
DCA: Deoxycholic acid
ECL: Enhanced chemiluminescence
ERK: Extracellular regulated protein kinase
ETEC: Enterotoxigenic *Escherichia coli*
FGF19: Fibroblast growth factor 19
FXR: Farnesoid X receptor
GCDCA: Glycochenodeoxycholic acid
GUDCA: Glycoursodeoxycholic acid
HCA: Hyocholic acid
HDCA: Hyodeoxycholic acid
HE: Hematoxylin and eosin
HEK293T: Human embryonic kidney cells 293
HRP: Horseradish peroxidase
IBABP: Ileal bile acid-binding protein
IBD: Inflammatory bowel disease
IECs: Intestinal epithelial cells
iFABP: Intestinal-type fatty acid binding protein
IL-1β: interleukin-1β
IL-6: interleukin-6
IKK: Inhibitor of kappa B kinase
IPEC-J2: Porcine small intestinal epithelial cells
IκB-α: Inhibitor of nuclear factor κB-α
LCA: Lithochalic acid
LPS: lipopolysaccharides
MEK: Mitogen-activated protein kinase
MRM: Multiple reaction monitoring
MRP2: Multidrug resistance-associated protein 2
MRP3: Multidrug resistance-associated protein 3
NF-kB: Nuclear factor kappa B
NLRP3: NOD-like receptor thermal protein domain associated protein 3
NTCP: Na^+^-taurocholic acid cotransport protein
OA: Oleanolic acid
OTUs: Operational taxonomic units
OST-α/β: Organosolute α/β heterodimer
pBD3: Porcine β-defense 3
PBS: Phosphate buffered saline
PCA: Principal component analysis
PCoA: Principal coordinate analysis
PKA: Protein kinase A
PMSF: Phenylmethanesulfonyl fluoride
PXR: Pregnane X receptor
p65: Transcription factor p65
RIPA: Radio immunoprecipitation assay
SDS-PAGE: Sodium dodecyl sulfate polyacrylamide gel electrophoresis
S1PR2: Sphingosine-1-phosphate receptor 2
SHP: Small heterodimer partner
TBST: Tris buffered saline with Tween-20
TGR5: Takeda G protein-coupled receptor 5
TNF-α: Tumor necrosis factor-α
UDCA: Ursodeoxycholic acid
UPGMA: Unweighted pair-group method with arithmetic means
VDR: Vitamin D receptor
ZO-1: Zona occludens 1
