# Supplementary material for: Oleanolic acid improved intestinal immune function by activating and potentiating bile acids receptor signaling in E. coli-challenged piglets
Source: J Anim Sci Biotechnol. 2024 May 18;15:79. doi: 10.1186/s40104-024-01037-0 (PMC11102245; doi:10.1186/s40104-024-01037-0)

| **Items** | **Content** | |
| --- | --- | --- |
| Diet ingredients,% | |  |
| Corn | 63.00 | |
| Soybean meal (45% CP) | 13.80 | |
| Corn gluten meal (55% CP) | 2.00 | |
| Full-fat soybean | 9.30 | |
| Dried whey | 3.00 | |
| Fish meal (67% CP) | 3.60 | |
| Soybean oil | 2.00 | |
| L-Lysine (98%) | 0.32 | |
| Dicalcium phosphate | 1.00 | |
| Limestone | 0.70 | |
| Salt | 0.28 | |
| Premix^a^ | 1.00 | |
| Total | 100.00 | |
| Nutrient levels^b^, % | | |
| ME, Mcal/kg | 3.31 | |
| Crude protein | 18.57 | |
| Crude fat | 6.61 | |
| Lysine | 1.25 | |
| Methionine | 0.35 | |
| Threonine | 0.73 | |
| Tryptophan | 0.21 | |
| Calcium | 0.76 | |
| Total phosphorus | 0.61 | |
| Available phosphorus | 0.38 | |
| Sodium | 0.18 | |
| Chlorine | 0.23 | |

**Table S1** Ingredients and compositions of the basal diet

^a^Provided the following per kilogram of diet: Fe (as ferrous sulfate), 190 mg; Cu (as copper sulfate), 190 mg; Mn (as manganese sulfate), 45 mg; Zn (as zinc sulfate), 140 mg; Se (as sodium selenite), 0.4 mg; I (as calcium iodate), 0.5 mg; vitamin A, 22,500 IU; vitamin D_3_, 4,250 IU; vitamin E, 40 mg; vitamin K_3_, 2.5 mg; vitamin B_1_, 4 mg; vitamin B_2_, 10 mg; vitamin B_6_, 4 mg; vitamin B_12_, 0.05 mg; niacin, 50 mg; D-pantothenic acid, 22.5 mg; D-biotin, 0.25 mg; and folate, 2 mg

^b^Crude protein, crude fat, calcium, and total phosphorus were analyzed values, the rest were calculated values

**Table S2** Sequences of primers used for real-time polymerase chain reaction

| **Target** | **Sequence (5’→3’)** | **Accession** |
| --- | --- | --- |
| β-actin | Forward: TTCCTTCCTGGGCATGGAGTCC | AJ312193.1 |
|  | Reverse: GGCGTACAGGTCTTTGCGGATG |  |
| TNF-α | Forward: GCTGATTTGGGTGACTAACTTG | JF831365.1 |
|  | Reverse: GCTTTTATTTCTCGCCACTGAC |  |
| IL-1β | Forward: CCCCAAAAGATACCCAAAGAG | NM_001302388.2 |
|  | Reverse: CTGCTTGAGAGGTGCTGATGATGTA |  |
| IL-6 | Forward: CCTCTCCGGACAAAACTGAA | AF518322.1 |
|  | Reverse: TCTGCCAGTACCTCCTTGCT |  |
| PMAP-23 | Forward: TATGAATTCCAGGCCCTCAGCTACAG | NM_001129976.1 |
|  | Reverse: TACGCGGCCGCCTTTGAACTCTTCCCTGTG |  |
| PMAP-36 | Forward: CTGGTCACTGTGGCTTCTGCTG | NM_001129965.1 |
|  | Reverse: GTGGGTCATTGGATGGGTTCAAGG |  |
| PG1 | Forward: CAGAGGAAGTCTTCTAACATGCG | NM_001123149.1 |
|  | Reverse: GCAGAACCTACGCCTACAAT |  |
| pBD-1 | Forward: TGCCACAGGTGCCGATCT | [NM_213838.1](https://www.ncbi.nlm.nih.gov/nuccore/NM_213838.1) |
|  | Reverse：CTGTTAGCTGCTTAAGGAATAAAG |  |
| pBD-2 | Forward: CCAGAGAGGTCCGACACT | [XM_021071238.1](https://www.ncbi.nlm.nih.gov/nuccore/XM_021071238.1)1 |
|  | Reverse: GGTCCCTTCAATCCTTGTAGGTGA |  |
| pBD-3 | Forward: CCTTCTCTTTGCCTTGCTCTT | NC_004818.2 |
|  | Reverse: GCCACTCACAGAACAGCTACC |  |
| pEP2C | Forward: ACTGCTTGTTCTCCAGAGCC | BK005522.1 |
|  | Reverse: TGGCACAGATGACAAAGCCT |  |
| PR-39 | Forward: AGCTGCAGTGACTCTCTTAAGGT | NM_214450.1 |
|  | Reverse: TCACCGCATGTTAGAAGACTTCC |  |
| Claudin-1 | Forward: AGAAGATGCGGATGGCTGTC | NM_001244539.1 |
|  | Reverse: CCCAGAAGGCAGAGAGAAGC |  |
| Occludin | Forward: TCCTGGGTGTGATGGTGTTC | NM_001163647.2 |
|  | Reverse: CGTAGAGTCCAGTCACCGCA |  |
| ZO-1 | Forward: AAGCCCTAAGTTCAATCACAATCT | XM_021098896.1 |
|  | Reverse: ATCAAACTCAGGAGGCGGC |  |
| CYP7A1 | Forward: AGGCTTCCCGATTCATGTGTTCAAG | XM_013996745.2 |
|  | Reverse: TCTGCGTCATCTAAGGTGGAGAGG |  |
| CYP8B1 | Forward: CAGGCAAGAAGATCCACCACTACAC | NM_214426.1 |
|  | Reverse: TGACCATGAGCAGCACAAAGAGC |  |
| CYP27A1 | Forward: ACTCACTCTACGCCACCTTCCTC | NM_001243304.1 |
|  | Reverse: GTATTCCAGCCATCCAGGTATCGC |  |
| FXR | Forward: TATGAACTCAGGCGAATGCCTGCT | NM_001287412.1 |
|  | Reverse: ATCCAGATGCTCTGTCTCCGCAAA |  |
| TGR5 | Forward: TGCTGTCCCTCATCTCATTGG | XM_021076201.1 |
|  | Reverse: TGTGTAGCGATGATCACCCAG |  |
| SHP | Forward: GCCTACCTGAAAGGGACCAT | AH014861.3 |
|  | Reverse: CAACGGGTGTCAAGCCTTTA |  |
| FGF19 | Forward: AAGATGCAAGGGCAGACTCA | XM_003122420.3 |
|  | Reverse: AGATGGTGTTTCTTGGACCAGT |  |
| IBABP | Forward: GCGACATAGAGACCATCGGG | NM_214215.2 |
|  | Reverse: GTAGTTGGGGCTGTTCACCA |  |
| NTCP | Forward: TTCCCTGCACCATAGGCATC | XM_001927695.5 |
|  | Reverse: CGAGCATTGAGGCGGAAAAG |  |
| OATP | Forward: CAGAAGATCCATCAGAGTGTGTGA | XM_021091164.1 |
|  | Reverse: GTGTTCACCGATCCAGTGTCA |  |
| OST-α | Forward: GACGGAGCCAGAAGGAAAGAC | NM_001244266.1 |
|  | Reverse: CAGACGGAGGGGATGCTGTA |  |
| OST-β | Forward: GGCGTGTGCTAAATGCAGAG | XM_005658570.3 |
|  | Reverse: GTTTTCCACACGGCTGTCAC |  |
| ASBT | Forward: CCAGAGTGCCTGGATCATCG | NM_001244463.1 |
|  | Reverse: GGAGTAACCGGCCAAAGGAA |  |
| BSEP | Forward: CGCAGCGTGAAGAAATGTGG | XM_003133457.5 |
|  | Reverse: AACCGAAACAGTTGAAAGAGGC |  |
| MRP2 | Forward: GGCTACTCCTGCGTGTTCTT | XM_021073710.1 |
|  | Reverse: TCCTCAGCAACATCCCACAC |  |
| MRP3 | Forward: GGTTGGAAGGCCACCGTTTT | XM_003131575.6 |
|  | Reverse: GTGTGCAAGGACAGGTTGGA |  |

**Table S**3 Effect of OA on productive performance of ETEC-infected piglets

| **Items** | **Groups** | | | | ***P*** |
| --- | --- | --- | --- | --- | --- |
|  | **CON** | **ETEC** | **ETEC + OA** | **OA** |  |
| Initial weight, kg | 11.39±0.46 | 11.34±0.30 | 11.21±0.31 | 11.66±0.39 | 0.848 |
| Final weight, kg | 20.36±0.91 | 18.59±0.53 | 18.19±0.44 | 19.88±0.58 | 0.065 |
| ADG, kg | 643.87±29.50^a^ | 544.72±19.01^b^ | 542.86±17.05^b^ | 637.50±28.35^a^ | 0.022 |
| ADFI, kg | 1210.06±36.34^a^ | 995.75±32.43^b^ | 1018.48±27.57^b^ | 1193.49±30.71^a^ | 0.001 |
| F/G | 1.89±0.08 | 1.87±0.06 | 1.90±0.06 | 1.86±0.08 | 0.976 |


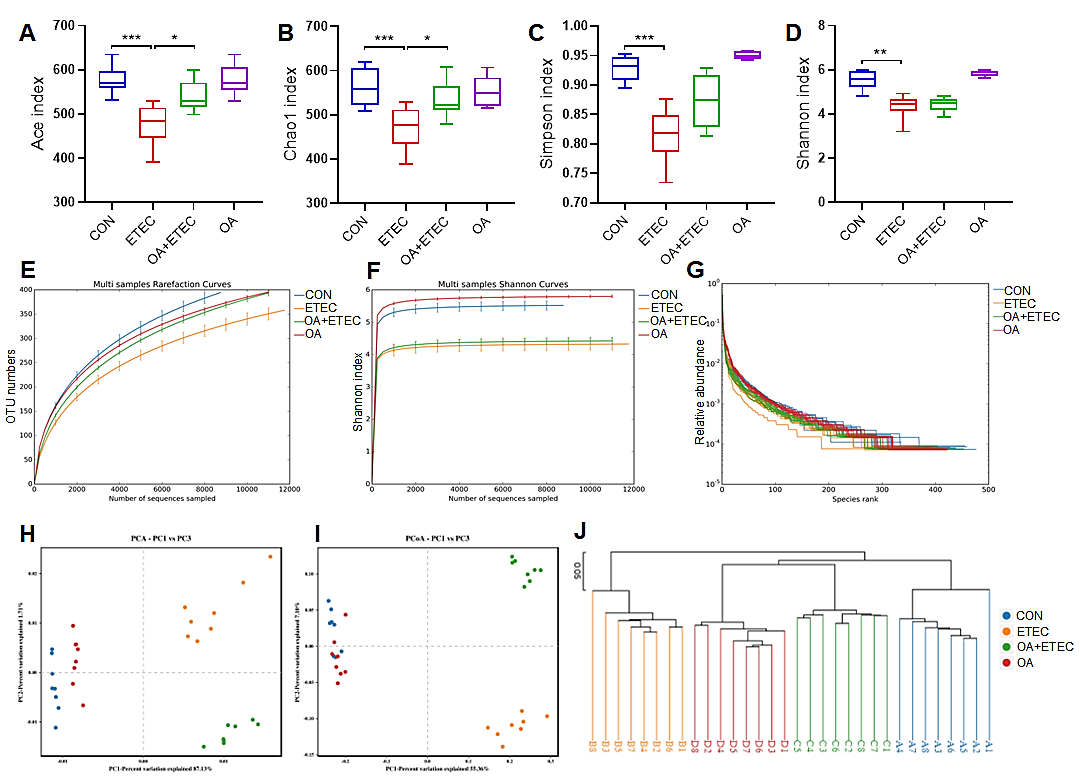


**Fig. S1** Effects of OA on the diversity, richness, and structure of gut microbiota in ETEC-challenged piglets. **A–D** Alpha diversity (Chao1 index, Ace index, Simpson index and Shannon index). **E–G** Shannon curves, rarefaction curves, and OTU rank curves for each group. **H–I** PCoA, and PCA score plot based on weights. **J** Weighted Unifrac cluster tree based on group method with arithmetic mean (UPGMA)


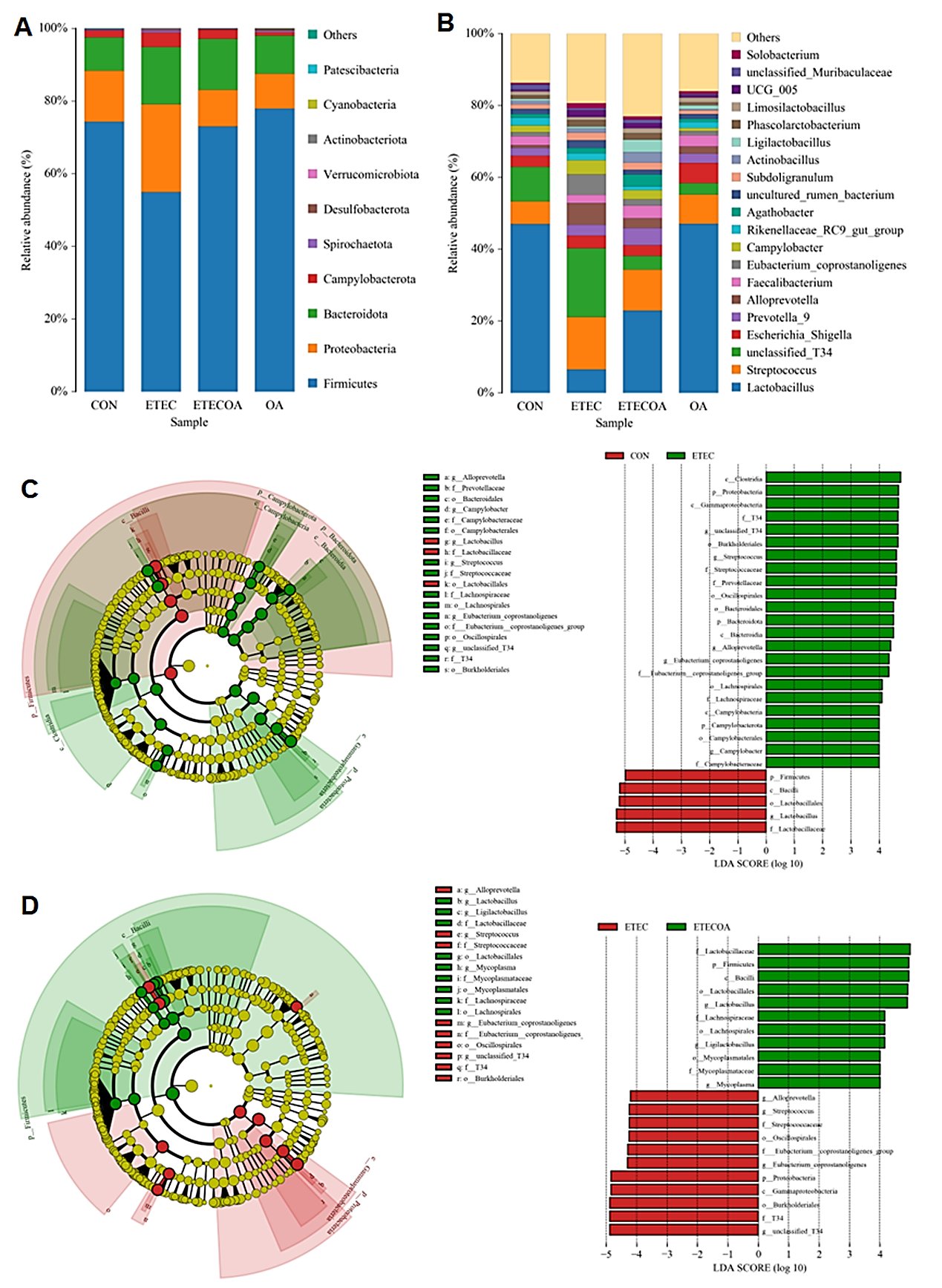


**Fig. S2** Compositions of cecum microbiota at the phylum (**A**) and genus (**B**) level. **C** and **D** The linear discriminant analysis (LDA) effect size was calculated to explore the taxa within genus levels that more strongly discriminated between the gut microbiota of mice fed with ETEC compared to CON group or ETEC+OA compared to ETEC

**Fig. S3** Total bile acids in the contents of the ileum and colon of piglets
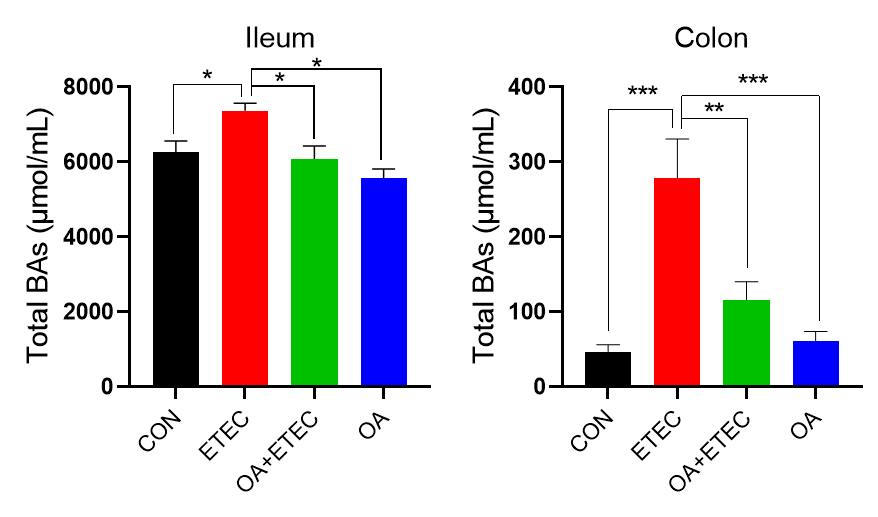

Supplement: Supplementary file 1 — Additional file 1: Table S1 Ingredients and compositions of the basal diet; Table S2 Sequences of primers used for real-time polymerase chain reaction; Table S3 Effect of OA on productive performance of ETEC-infected piglets; Fig. S1 Effects of OA on the diversity, richness, and structure of gut microbiota in ETEC-challenged piglets; Fig. S2 Compositions of cecum microbiota at the phylum (A) and genus (B) level. C and D The linear discriminant analysis (LDA) effect size was calculated to explore the taxa within genus levels that more strongly discriminated between the gut microbiota of mice fed with ETEC compared to CON group or ETEC+OA compared to ETEC; Fig. S3 Total bile acids in the contents of the ileum and colon of piglets. [file 40104_2024_1037_MOESM1_ESM.docx]
